# *eHealth Familias Unidas Mental Health*: Protocol for an effectiveness-implementation hybrid Type 1 trial to scale a mental health preventive intervention for Hispanic youth in primary care settings

**DOI:** 10.1371/journal.pone.0283987

**Published:** 2023-04-18

**Authors:** Yannine Estrada, Alyssa Lozano, Devina Boga, Maria I. Tapia, Tatiana Perrino, Maria Rosa Velazquez, Lourdes Forster, Nicole Torres, Cecilia V. Morales, Lisa Gwynn, William R. Beardslee, C. Hendricks Brown, Guillermo Prado

**Affiliations:** 1 School of Nursing and Health Studies, University of Miami, Coral Gables, FL, United States of America; 2 Department of Public Health Sciences, Miller School of Medicine, University of Miami, Miami, FL, United States of America; 3 UMMG UHealth—Kendall, Miller School of Medicine, University of Miami, Miami, FL, United States of America; 4 UMMG, Clinical Pediatrics, Miller School of Medicine, University of Miami, Miami, FL, United States of America; 5 Department of Psychiatry, Boston Children’s Hospital, Boston, MA, United States of America; 6 Harvard Medical School, Harvard University, Cambridge, MA, United States of America; 7 Department of Psychiatry and Behavioral Sciences, Northwestern University, Chicago, IL, United States of America; 8 Department of Preventive Medicine, Northwestern University, Chicago, IL, United States of America; 9 Department of Medical Social Sciences, Northwestern University, Chicago, IL, United States of America; UNITED KINGDOM

## Abstract

This article focuses on the rationale, design and methods of an effectiveness-implementation hybrid type I randomized trial of *eHealth Familias Unidas Mental Health*, a family-based, online delivered intervention for Hispanic families to prevent/reduce depressive and anxious symptoms, suicide ideation/behaviors, and drug use in Hispanic youth. Utilizing a rollout design with 18 pediatric primary care clinics and 468 families, this study addresses intervention effectiveness, implementation research questions, and intervention sustainment, to begin bridging the gap between research and practice in eliminating mental health and drug use disparities among Hispanic youth. Further, we will examine whether intervention effects are partially mediated by improved family communication and reduced externalizing behaviors, including drug use, and moderated by parental depression. Finally, we will explore whether the intervention’s impact on mental health and drug use, as well as sustainment of the intervention in clinics, varies by quality of implementation at clinic and clinician levels.

**Trail registration:** ClinicalTrials.gov Identifier: NCT05426057, First posted June 21, 2022.

## Introduction

Approximately 15% of Hispanic youth between 12–17 years reported having a past year major depressive episode (MDE), a steadily increasing rate compared to the previous three years and the highest rate of all other Hispanic age groups [1]. Mental health disorders developed during adolescence are associated with later negative health outcomes, including reoccurrence of depression and/or anxiety in adulthood [2, 3], anxiety disorders, bipolar disorder [4], suicide attempts, alcohol/drug dependence, and unsafe sexual behaviors [5]. Notably, drug use behaviors, including illicit drug, marijuana, opioid, risky alcohol, and cigarette use, were more prevalent among adolescents who reported a past year MDE than their adolescent counterparts without a past year MDE [6].

Family functioning behaviors, such as family communication, may reduce and/or prevent poor mental health and drug use outcomes in adolescents. For example, positive family relations have been negatively associated with suicide risk among adolescents [7]. Conversely, poor family communication may have adverse effects on adolescents’ mental health [8, 9]. Parent-adolescent communication, another dimension of family functioning, has been shown to be negatively associated with both internalizing and externalizing behaviors (e.g., drug use) in adolescents [10]. It is possible that for certain youth, family functioning protects against poor mental health outcomes indirectly by reducing externalizing behaviors, including drug use behaviors [11].

The association between family functioning (i.e., communication) and both mental health and drug use suggest that family-based interventions may be a viable solution for prevention. In fact, family-based interventions developed to address drug use prevention have shown crossover effects in reducing the prevalence of mental health symptoms among adolescents [7, 12, 13]. Despite the effectiveness of these interventions, few have been tailored to meet the needs of Hispanic families. Family plays a critical role in either mitigating or amplifying adolescent health outcomes such as poor mental health and drug use among adolescents. For Hispanic adolescents in particular, family functioning is especially pertinent due in part to Hispanic cultural values related to commitment, loyalty, and obligation felt towards the family system [14]. One intervention which has been culturally tailored to address Hispanic adolescent drug use and sexual risk behaviors by targeting family functioning, including family communication, is *Familias Unidas* [15]. For families with poor communication, Familias Unidas has shown crossover effects in lowering internalizing symptoms [16], externalizing symptoms [17], and in reducing suicidal attempts [18]. Importantly, these outcomes occurred through improvements or changes in parent-adolescent communication. Further, the online adaptation of Familias Unidas, *eHealth Familias Unidas* [19], demonstrated effects in reducing drug use and improving family functioning [20].

Despite their efficacy, few evidence-based preventive interventions (EBPIs) such as Familias Unidas and eHealth Familias Unidas, have been implemented outside of research settings. An entry point to broaden the reach of preventive services for youth are pediatric primary care settings [21, 22], they hold remarkable potential for the implementation of EBPIs to promote mental health for several reasons. First, primary care settings are visited often by adolescents. In 2020, approximately 94.5% of Hispanic adolescents under the age of 18 reported seeing a doctor or another health care professional during the previous 12 months [23]. Second, primary care doctors are considered a trusted source of health information, such that parents often consult primary care providers for adolescent behavioral concerns [22]. Finally, primary care settings may provide a solution for the underutilization of mental health services by de-stigmatizing access to care and increasing the probability of mental health screenings. Given these reasons, primary care settings may, therefore, be a viable real world setting to evaluate EBPIs that target the mental health and drug use disparities seen in Hispanic populations [24].

Evaluating EBPIs in real world settings is critical for understanding the implementation processes that facilitate or impede incorporation of such interventions into different systems such as primary care clinics. Integration of EBPIs in real world settings increases reach for those in need [25]. However, adoption and success of EBPIs may vary as a function of different implementation factors such as clinic organization, organizational climate for evidence-based implementation, and clinic leadership [26–28]. For example, organizational change, such as introducing an EBPI into a primary care clinic, may be limited or facilitated by provider attitudes toward adoption of new interventions and practices [29, 30].

As interventions continue to move through translational stages, understanding how various organizational factors impact the implementation process may be used to inform subsequent implementation stages [31] in addition to improving understanding of sustainment in real world settings [32, 33]. Further, it is also critical to ensure that EBPIs are effective in real world settings when delivered by non-research personnel. Effectiveness-implementation hybrid type I designs allow researchers to simultaneously address clinical effectiveness and implementation research questions and outcomes [34]. With these designs, researchers can concurrently evaluate clinical interventions while gathering information on delivery, implementation, and sustainment of the intervention in real-world practice [34]. This article describes the rationale, design, and methods of an effectiveness-implementation hybrid type I randomized trial of eHealth Familias Unidas Mental Health, an online delivered intervention for Hispanic families to prevent/reduce depressive symptoms, anxiety symptoms, suicide ideation/behaviors, and drug use in Hispanic youth. Utilizing a rollout design with 18 pediatric primary care clinics, this study addresses both effectiveness and implementation research questions, and examines sustainment of the intervention, the least studied area of implementation science [33, 35]. We hypothesize that eHealth Familias Unidas Mental Health will be effective, relative to standard of care, in reducing elevated levels of depressive symptoms, anxiety symptoms, suicide ideation, suicide attempts, and drug use. Further, we will examine whether intervention effects are partially mediated by improvements in family communication and reductions in externalizing behaviors, including drug use, and moderated by parental depression. Finally, we will explore whether the intervention’s impact on mental health and drug use, as well as sustainment of the intervention in clinics, varies by quality of implementation at clinic and clinician levels.

### RE-AIM framework

This study will be guided by the RE-AIM Framework [25] a comprehensive and well-established framework used to evaluate the impact of health promotion interventions. The RE-AIM framework utilizes five dimensions to evaluate programs: reach, efficacy, adoption, implementation, and maintenance. Further, these dimensions are assessed at multiple levels (e.g., the individual and organizational level) and interact to yield the public health impact of a health intervention [19]. Specifically, in this study, RE-AIM will be used to evaluate: the percentage and characteristics of participants that engage in the intervention (individual level reach dimension); the effectiveness of eHealth Familias Unidas Mental Health (efficacy dimension) on outcomes including depressive symptoms, anxiety symptoms, suicide ideation and behaviors, as well as organizational level changes along with any negative study outcomes at the organizational and individual level; adoption of the intervention will be measured based on the proportion and representation of clinic type (e.g., private vs. public) that adopt the intervention as well as the characteristics of the clinics where the intervention is delivered (adoption dimension); implementation assessments will be based on fidelity measures that evaluate how well the intervention was delivered as originally intended (implementation dimension); maintenance will be evaluated based on the proportion of clinics that sustain the intervention after the research team ends involvement with the clinic (maintenance dimension).

## Methods: Participants, interventions, and outcomes

**Fig 1:**
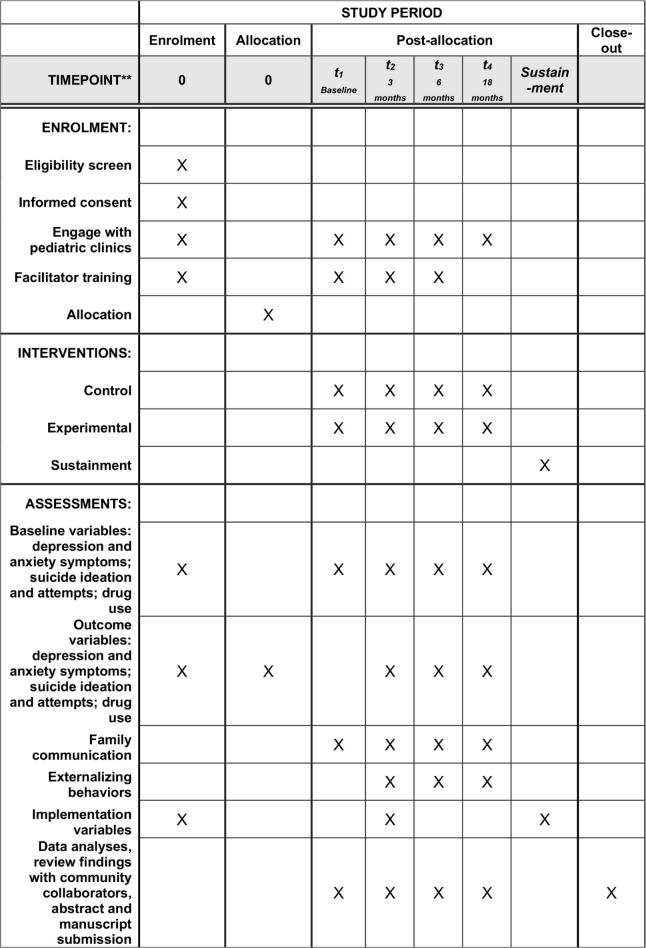
Schedule of enrolment, intervention, and assessments.

This study was approved on May 4^th^ 2021, by the University of Miami Human Subjects Research Office, study # 20210286. All participants complete written informed consent and assent before data collection. The schedule of enrolment, intervention, and assessments is available in Fig 1. The study is actively recruiting and enrolling participants, during this manuscript preparation, from the South Florida area in the U.S. The study team followed clinicaltrials.gov guidelines, which require that clinical trial information be submitted no later than 21 days after the first participant is enrolled. The authors confirm that all ongoing and related trials for this intervention are registered. Recruitment is scheduled to last 15 months from April 2022 and follow-ups will run through December 2024.

### Study design

**Fig 2:**
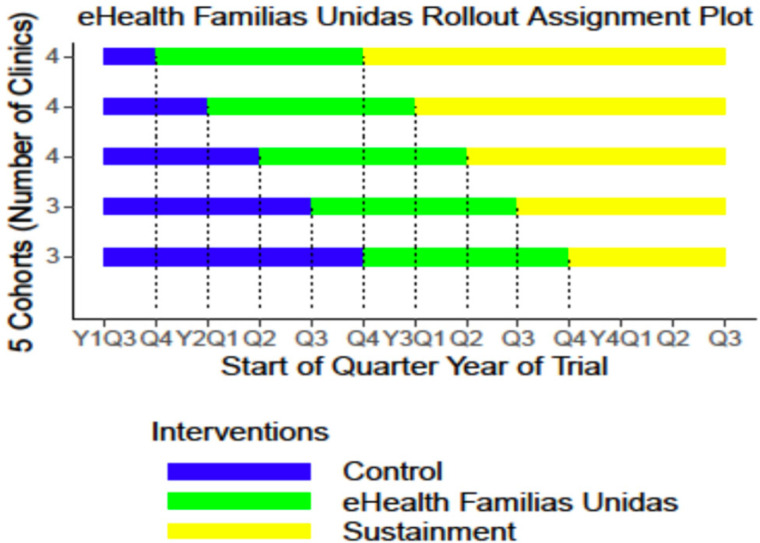
eHealth Familias Unidas rollout assignment plot.

To evaluate eHealth Familias Unidas Mental Health in an effectiveness-implementation hybrid Type 1 trial, we will use a rollout design, an extension of a Stepped Wedge design [36], with three conditions (control, experimental, and sustainment) and both within and between clinic comparisons of effectiveness over time. We will balance on multiple clinic-level variables including zip code, percentage of Hispanic patients, number of primary care physicians, presence of mental health services, and whether the clinic has FQHC (federally qualified health center) status. Then 18 pediatric primary care clinics will be randomized to five consecutive cohorts of sizes 4, 4, 4, 3, and 3 (Fig 2). Each cohort will be staggered one quarter year apart. The clinics are heterogeneous and consist of federally qualified health centers, academic primary care clinics located within the University of Miami’s Health District, and private community clinics. A list of clinics can be found at ClinicalTrials.gov.

The randomization variable for this study is time. That is, we are randomizing *when* each clinic begins to deliver the intervention to participants. All clinics begin with a control phase during which the intervention is not delivered, followed by the experimental phase during which clinic staff deliver the intervention, and conclude with a sustainability phase. The control condition consists of the regular practices that clinics implement to address mental health symptoms (e.g., screening, referral). We chose this control to be consist with what is practiced in a real-world setting. During the sustainability phase, the research team no longer provides formal support (i.e., clinical supervision) to the clinic in delivering the intervention.

### Participants and inclusion criteria

The sample will include 468 Hispanic youth and their primary caregivers. The inclusion criteria consists of: (a) youth who self-identify as Hispanic/Latino(a), (b) youth between 12–16 years old, (c) youth living with an adult primary caregiver who is willing to participate, (d) families must have access to broadband internet on a device, including (but not limited to) a smartphone, iPad, tablet, computer at home or other location (e.g., school, library, etc.), (e) parent reported low family functioning; or youth reported elevated depressive symptoms; or youth reported anxiety symptoms; or a history of suicide behavior (ideation or attempts). Families with plans to leave South Florida during the study period are excluded from the study.

### eHealth Familias Unidas Mental Health intervention

The eHealth Familias Unidas Mental Health facilitators will consist of staff within the 18 primary care clinics. Facilitators may include nurse assistants, mental health counselors, or any other health professional located within the participating clinics. The requirements to participate as a facilitator include: 1) be bilingual/bicultural, 2) have a bachelor’s degree, 3) be willing to deliver the intervention, and 4) have permission from respective supervisors. The experimental condition consists of eHealth Familias Unidas Mental Health, an adaptation of the eHealth Familias Unidas intervention [21]. eHealth Familias Unidas Mental Health aims to promote adolescent behavioral health, including the prevention of drug use, by improving family communication. The intervention also includes components that directly target internalizing (depression and anxiety) and suicide symptoms. The intervention is based on ecodevelopmental theory [37, 38], a contextual, multi-level framework that provides a useful way to organize and address risk and protective factors for youth drug use, externalizing behaviors, and internalizing symptoms from the macrosystem (i.e., the broad societal factors and philosophical ideals that define a particular culture—e.g., Hispanic cultural values and norms) to the microsystem (i.e., the contexts in which youth participate directly—e.g., family, peers). Ecodevelopmental theory helps frame prevention intervention efforts by addressing the multiple risk and protective factors operating at different levels of youth’s social contexts that influence adolescent health. The eHealth Familias Unidas Mental Health intervention consists of 14 sessions (see Table 1): nine (20–40 minute) video parent group sessions in Spanish (with English subtitles), one (30 minute) video adolescent group session, and four (45-minute) web-conferenced family sessions delivered in either Spanish and/or English, depending on parental level of comfort with either language.

**Table 1:** eHealth Familias Unidas Mental Health–Session by session overview.

Session	Description
**Online Family Session #1 Engagement Family Session**	Completion of family needs assessment and problem-solve family’s perceived barriers to participation. Engage family in the multiple intervention goals of promoting youth behavioral well-being, including: prevention of drug use, externalizing behaviors, sexual risk, internalizing symptoms and suicide through strengthening family and interpersonal skills and relationships.
**Parent Video Group Session #1 Parental Investment in Adolescent Worlds**	Provides an introduction to eHealth Familias Unidas and a review of adolescent risk factors. Parents engage in an interactive exercise to set goals in each of the adolescent’s worlds.
**Parent Video Group Session #2 Enhancing Communication Skills**	Focuses on the characteristics of effective family communication and parents engage in an interactive exercise to reinforce key communication skills.
**Parent Video Group Session #3 Mental Health and Family**	Parents learn about identifying internalizing and suicide symptoms, as well as risk and protective factors. They apply family communication skills relevant to mental health promotion, including listening and monitoring youth mental health, promoting positive parent-adolescent relationships, and reducing isolation/ enhancing social support. Focus is also on screening for parent mental health symptoms, the value of parent self-care and well-being, how parent mental health affects families, parenting and adolescent wellness. Coping mechanisms are covered (e.g., social support), and resources.
**Adolescent Video Session #1 Introduction to understanding Depression in Adolescents**	Youth engage in interactive online modules to learn and practice mental health promotion, with focus on family and interpersonal skills (e.g., communication, seeking social support) to address risks (e.g., family conflict, peer rejection or bullying, isolation). Sessions involve screening for mental health and suicide symptoms with referral to care for those showing clinically-relevant symptoms. Resources available in the community are provided.
**Online Family Session #2 Family Communication**	Facilitator meets with family so that the parents and adolescents can exercise newly learned communication and interpersonal skills & practice together by discussing a relevant issue in the youth’s life.
**Parent Video Group Session #4 Family Support and Behavior Management**	Highlights the significance of parental support, behavior management, and effective discipline. Interactive exercise reinforces behavior management strategies. Discuss the role of effective behavior management skills on adolescent mental health.
**Parent Video Group Session #5 Parental Monitoring of Peers**	Highlights the role of prosocial and antisocial peers and the protective effects of monitoring. Parents complete an interactive exercise that reinforces parental monitoring strategies. Discuss how monitoring adolescents’ relationships help them address mental health/suicide risks (e.g., adolescent isolation, peer rejection, conflict, bullying).
**Parent Video Group Session #6 Adolescent Substance Use: Attitudes, Beliefs, Intentions, & Peer Pressure**	Focuses on the prevalence and consequences of adolescent drug use. Parents complete an interactive exercise on strategies to prevent adolescent drug use. The bidirectional connection between drug/ alcohol use and depression, anxiety, and suicide is emphasized, as well as common family and interpersonal protective factors parents have learned about in other sessions.
**Online Family Session #3 Parental Monitoring of Peer World and Adolescent Drug Use**	Covers family conversations about adolescent’s peers and ways to troubleshoot interactions between parents and the youth’s peer world. Parents teach youth the skills necessary to effectively manage peer pressure to engage in drug use. Discuss the role of interpersonal peer relations in exacerbating or protecting from mental health and suicide risks, with parents guiding youth to enhance positive relations.
**Parent Video Group Session #7 Parental Investment in School**	Addresses the role of school in the adolescent’s life and how parental connections to school can serve as a protective mechanism. Parents engage in an interactive exercise that emphasizes parental involvement in the adolescent’s school world.
**Parent Video Group Session #8 Adolescent Risky Sexual Behavior and HIV: Attitudes, Beliefs, Intentions, & Peer Pressure**	Discusses parental attitudes and beliefs regarding adolescent sexual risk behaviors, the effects of adolescent sexual risk behaviors on STIs, and what parents can do to influence adolescent behavior. Parents complete two interactive exercises to reinforce knowledge of risky sex and safe sex practices.
**Online Family Session #4 Adolescent Sexual Risk Behaviors**	Parents communicate the dangers and consequences of risky sexual behavior. Parents guide their adolescent in developing safety skills. Discuss the co-occurrence of mental health and sexual risk behaviors in youth and ways to protect from both.
**Parent Video Group Session #9 Prevention Has to Be Achieved All Over Again Everyday**	Highlights parents’ role as lifetime educators of their adolescent and the importance of daily implementation of skills to improve family functioning, including family communication, in order to prevent drug use, sexual risk behaviors, and mental health risks. Parents watch a review of the interconnectedness among these behaviors and the importance of parental involvement, family communication, family support, and parental monitoring in combating these risks.

The video parent group sessions consist of 1) a culturally syntonic telenovela/soap opera series, 2) videotaped parent group discussions, and 3) interactive exercises. The youth online session addresses interpersonal and social protective factors for youth internalizing and suicide symptoms. The session highlights the importance of parental positive support and peer relations. The sessions educate youth and parents about mental health symptoms and suicide. The mental health specific sessions build heavily upon the existing family communication session to highlight how this proven protective factor provides opportunities for listening and monitoring youth mental health, as well as providing support for the adolescent such as positive and supporting family relations. Importantly, the parent session addresses parent self-care, how parent mental health affects family functioning, including family communication, parenting, and, ultimately, adolescent well-being. These components are culturally consonant for Hispanic families, allowing reinforcement of family interconnectedness and the role of parents as competent family leaders. These values are congruent with the core aspects of the intervention, including mechanisms of action, such as promoting family communication. In the online family sessions, the facilitator and one family (i.e., adolescent and parent(s), and/or others significantly involved in parenting the youth) participate together and provide parents the opportunity to practice with their youth the acquired skills from the video parent group sessions.

### eHealth Familias Unidas Mental Health facilitator training, supervision, and fidelity

Facilitators are responsible for tracking the families’ progress in watching the video parent group sessions, delivery of four online family sessions, and participating in the eHealth Familias Unidas Mental Health training and supervision. Facilitators are paid to complete training, supervision, and delivery of the intervention. Facilitators also complete assessments regarding sustainment and implementation and are paid $20 for completing these measures.

Training and supervision for eHealth Familias Unidas Mental Health is performed by the study clinical trainer/supervisor. Training is a total of 16 hours delivered in two 1-day workshops. Supervision consists of 1-hour weekly meetings across 12 weeks. Training approaches include review of the intervention manual, didactic presentations, videotape reviews and role-plays between the primary care facilitator and the clinical supervisor. Facilitators are also trained on strategies to build strong alliances with families. Additionally, two-hour group supervision sessions for each of the four family sessions include didactic presentations, case review and planning for upcoming sessions, and review of fidelity ratings. To monitor implementation fidelity, 25% of all videotaped family sessions conducted by each facilitator are randomly selected and rated by an independent adherence rater. To obtain inter-rater reliability, our lead fidelity trainer rates 25% of the family sessions rated by the independent rater. The clinical supervisor reviews fidelity ratings with the facilitators during group supervision sessions to identify and problem-solve fidelity concerns.

### Engaging clinic staff

The study clinical supervisor is responsible for establishing and maintaining relationships with the primary care clinics. Engaging and maintaining contact with the study’s primary care clinics is crucial. The clinical supervisor makes the initial contact with senior administrators within the clinics to explain the study and begin establishment of a collaborative relationship with the study team. After obtaining buy-in from senior administrators, there is an initial team meeting with clinic staff and research study staff. During this meeting, the research team provides information about the study and emphasizes the importance of a collaborative relationship between the clinic and research teams to prevent mental health symptoms and drug use among Hispanic youth. Additionally, the study team discusses with clinic personnel the importance of understanding clinic characteristics that promote the adoption and sustainment of evidence-based interventions.

Clinic personnel are involved, as much as possible, in study decision making such as study processes and procedures. For example, input is sought from clinic personnel on recommendations for recruiting participants, preferred ways to maintain/sustain contact with clinic personnel, and how to best integrate the study into the clinic flow. Maintenance of steady contact between primary care site teams and the research team is essential for the success of the study. Accordingly, frequent contact with primary care providers is encouraged to problem solve challenges that may arise including issues with recruitment, facilitators, and reporting of study progress to administrators.

### Data collection and recruitment

Families are assessed at baseline, 3-, 6- and 18- months post-baseline. Parent participants receive $40, $45, $50, and $55, respectively, at each assessment timepoint. Adolescent participants receive two movie tickets and $20 at each assessment. All consent, assent and questionnaire measures are available in Spanish and English. All surveys (i.e., screening and study assessments) are programmed and administered using HIPAA-compliant REDCap software [39] except for one questionnaire using computer adaptive testing (K-CAT® and CAT-MH®) with an embedded link in REDCap.

Once a family expresses interest in study participation, the parent and adolescent are each handed a separate tablet/iPad to fill out a screening questionnaire. The screening criteria scores are calculated utilizing REDCap programming and populated into form for interpretation by trained assessors. Assessors then confirm the criteria is met and move onto the consenting and assenting procedures. Following the consent and assent procedures, parents and adolescents complete the baseline assessment. Baseline measures are collected in the primary care clinic and follow-up survey links are sent to participants via email directly from REDCap. The study assessments include the CAT-MH®, a suite of adaptive tests that have been validated against structured clinical diagnostic interviews (i.e., DSM and SCID) using multidimensional item response theory [40]. Alerts have been enabled for suicide questions that are endorsed by the adolescent or by the parent regarding the adolescent in the screener and main survey; these alerts are received by trained study mental health professionals who follow up with the adolescent and the parent to triage for suicide risk and make referrals as needed. Alerts have also been put in place if there is increased mental health symptoms and/or drug use. The study has a scientific advisory board as well as a data safety and monitoring board (DSMB), and data safety and monitoring plan (DSMP). In the event that a participant should be withdrawn from the study, this will be consulted study with mental health professionals as well as the DSMB and reported to appropriate entities (e.g., IRB). Further, data quality checks are conducted on a monthly basis by the study research team and study procedures have been operationalized and documented. Any study violations, adverse events, and serious adverse events are reported to the IRB, funding agency (SAEs), and DSMB.

To protect confidentiality, two different ID mechanisms are in place, one generated by the REDCap system and a second ID created by the study team and assigned to each family, the assessment data are kept separately from any identifying information. Only approved personnel have access to ID information. All data are electronically stored in a secure university server after transfer from REDCap. Passwords are used to restrict entry into the database. Project staff, including the clinic facilitators, are trained on issues of confidentiality. Study assessors are present to clarify any questions or problem solve issues that may come up during the assessments. If research personnel are not available in the clinics when potential participants may be interested in the study, a flyer, along with contact information is provided, and assessors follow up with the families to screen and potentially enroll the family electronically. Additionally, families can be connected via protected videoconference software to reach the research team via an iPad. The team member will explain the study to and complete an e-consent process. Families who enroll will then be sent a link to complete the assessment battery on the iPad while they wait to see the physician. Consistent with prior trials, we will collect participant address, email and telephone where participants can be contacted for follow-up. Participants will also complete a detailed tracking form that includes three secondary contacts (e.g., family, friends) if we are unable to make contact for follow-up assessments. In the event of a missed assessment timepoint, the participant will be contacted for the following assessment. The data from this study will be available through the NIMH Data Archive.

#### Measures

All data are self-reported. The main outcomes of this study include adolescent depressive symptoms, anxiety symptoms, youth suicide ideation and behavior, and youth drug use. Family functioning indicators and youth externalizing problems are examined to determine whether they serve as mediators between condition and intervention outcomes. Parent depression is examined to determine whether it is a moderator between condition and intervention outcomes. As previously mentioned, clinic level data is being collected to assess implementation and sustainment, personnel and facilitators from each clinic will complete self-report surveys through REDCap regarding attitudes towards evidence-based practices, leadership, climate, and sustainability after clinic randomization, after delivery of the intervention has been completed, and at the end of the sustainability phase (i.e., end of the trial).

*Screener*. The parent screener to determine eligibility includes six demographic questions and the Parent-Adolescent Communication Scale (PAC) [41]. The adolescent screener includes two demographic questions, the Center for Epidemiologic Studies Depression Scale (CESD) [42], the Generalized Anxiety Disorder measure (GAD-7) [43], and two questions pertaining to lifetime suicide ideation and suicide attempt utilizing the Suicidal Behavior Questionnaire-Revised [44] and the Suicidal Ideation Questionnaire [45].

#### Sociodemographic characteristics

Demographic information is collected from both the parent and adolescent: age, date of birth, country of birth, marital status, gender identity, race/ethnicity, employment status, and family income. Additionally, information regarding mental health and other service utilization is collected from the parent and youth [46]. Participation in other services does not preclude participant study involvement.

#### Mental health

Depressive and anxiety symptoms are assessed with multiple questionnaires. Both parent and adolescent receive the DSM-5 Level 1 Cross-Cutting Symptom Measure which assesses multiple mental health domains including depression, anxiety, mania, and anger [47]. The Revised Children’s Anxiety and Depression Scale (RCADS-25) includes 25 questions that ask the parent about depressive and anxiety symptoms that they observe in their child and for the child it is self-reported [48]. The Patient-Health Questionnaire (PHQ-9) is a self-report measure administered to parents and youth consisting of 9 items that describe depressive symptoms and ask about how often the participant was bothered by experiencing the problems (for the parent it measures their own levels of depressive symptoms) [49]. The Generalized Anxiety Disorder-7 (GAD) is given to parents and youth as a self-report with 7 questions pertaining to anxiety symptoms over the last two weeks [43]. The DSM-5 Cross-Cutting Symptom measure, GAD, PHQ-9, and RCAD-25 are included to bolster standardization of mental health measurement across studies nationwide. Two computer adaptive technology measures are included: CAT-MH® for adults (to report on adolescent symptoms) and the K-CAT® for youth [50].

#### Drug use

Adolescent drug use is measured with the *Monitoring the Future Survey* [51]. This 47-item self-report instrument assesses lifetime drug use, type of drug use, frequency of each drug (with or without prescription) in the past 7 days, 1-month, and 3-months.

#### Externalizing behavior

The Revised Behavior Problem Checklist (RBPC) [52] includes an externalizing subscale of fifty-five items that assesses parent perceptions of adolescent problem behaviors.

#### Family functioning

Family functioning measures include questions pertaining to parent-adolescent communication, parental practices, and parental monitoring. The Parenting Practices Questionnaire includes two subscales of positive parenting (9-items) and parental involvement (20-items) [53]. Communication is measured with the Parent Adolescent Communication scale which consists of 20 items [41]. Parents and youth also report on parental monitoring with 5-items that focus specifically on monitoring of youth and time spent with their peers [54].

*Implementation and sustainment measures*. Three measures will assess experiences of clinic personnel regarding factors such as acceptability, attitudes, and prioritization of implementation. The Organizational Change Recipients’ Beliefs Scale (OCRBS) includes 24 items and measures the acceptability, appropriateness, and feasibility of delivering eHealth Familias Unidas Mental Health by clinic personnel [26]. The Evidence-Based Practice Attitude Questionnaire (EBPAS) examines clinic personnel’s attitudes toward adoption of evidence-based practices [29]. The Implementation Climate Scale (ICS) (18 items) [55] measures the extent to which clinic personnel feel their organization prioritizes and values the successful implementation of eHealth Familias Mental Health Mental Health. Two measures assess leadership in the organizational setting: The Implementation Leadership Scale (ILS) and Multifactor Leadership Questionnaire (MLQ) [27, 56]. The ILS consists of eleven items and assesses whether clinic personnel feel supported by clinic leadership to implement eHealth Familias Unidas Mental Health in their practice and to assess how well leaders rate their own ability to implement the eHealth Familias Unidas Mental Health in their practice. The MLQ includes thirty items and assesses clinic leaders transformational and transactional leadership behavior. Sustainability is measured using the Sustainment Measurement System Survey (SMS) which consists of fifty-three items that is completed by clinic leaders [33]. The SMS measures the sustainability of eHealth Familias Unidas Mental Health following the formal year-long implementation period.

### Data analysis plan

#### Data preparation and preliminary analyses

Statistical tests for univariate and multivariate normality (skewness and kurtosis), as well as visual inspections of the empirical distributions for the data are conducted at each time point to test distributional assumptions [21]. In the presence of concerning deviations, transformation of variables will be attempted when possible. Based on prior studies, the distribution of our outcomes will be skewed, often exhibiting a Poisson distribution. Therefore, we will utilize a Poisson regression [57, 58] or a two part growth curve model [59] in the event of positively skewed distributions. For all scale scores, we will generate reliability estimates of internal consistency (Cronbach’s alpha). Item total correlations and factor analyses will be used in instances of reliability estimates lower than .80 [60]. To account for multiplicity adjustments in the proposed analyses described below, we will use sensitive analyses for primary outcomes.

*Measurement modeling*. To evaluate the effectiveness of eHealth Familias Unidas Mental Health in preventing/reducing depressive and anxiety symptoms; suicide ideation and attempts; and drug use, we will analyze impact using growth curve modeling with four time points modeled as a multivariate normal distribution. For our binary outcomes (i.e., suicide ideation and attempts) and drug use we will conduct generalized growth modeling with mixed effects using these repeated binary measures. For our standard analyses we will use M*Plus* [61] to examine changes in growth using two-level modeling of clinic and individual, commonly called three-level modeling in the mixed effect modeling literature (individuals with repeated measures nested within clinic) [62, 63]. Since youth are recruited from all 18 clinics both during the control and experimental phases, we can consider clinic to be a “blocking factor,” therefore, the overall effect is essentially a mean of difference in mean slope parameters for eHealth Familias Unidas Mental Health versus control assigned participants. Base rates for suicide attempts are low and thus we account for them in our analytic plan and power analyses. In a universal trial of Familias Unidas, we found that at baseline, 9.2% of youth reported suicide ideation and 5.7% reported a suicide attempt in the preceding year. We present power analyses below based on these low, but not insignificant, rates. Finally, to ensure that our final timepoint, which has a longer time gap, does not overly influence the findings, we will examine transformation of the time axis [64]. After suitable transformation of the time axis, handling of missing data, and model fit checking, we will use a Wald type test on the difference in mean slope parameters for eHealth Familias Unidas Mental Health versus control assigned participants.

For mediation analyses, we will use the “product of coefficient” approach [65] to test for an indirect effect of improved family communication by 3 months, decreased externalizing behaviors by 6 months, and further decreased depressive and anxiety symptoms as well as suicide ideation and attempts by 18 months. We analyze this in 2 parts, with family communication affecting externalizing followed by externalizing behaviors affecting depressive symptoms. For the second model, the product of the effect of family communication on intervention (a coefficient) and depression on family communication controlling for intervention (b coefficient) measures this latter indirect effect. We will use M*plus* to compute bootstrapped confidence intervals since this product has a very non-normal distribution. Similarly, we will examine whether changes in externalizing behaviors (including drug use) at 6 months mediate the 18-month depressive, anxious, and suicide behavior outcomes. In addition, we propose to test whether the slope of externalizing behaviors changes on 18-month outcomes as well.

To examine moderation by parental depression, we will test for an interaction with that symptomatology at baseline and intervention, both in growth models and in 18-month outcomes. Given that parental depression can significantly impair a person’s ability to parent as well as their family functioning [66] and past prevention programs have found that parent depression moderates intervention effects in this manner [67], we hypothesize that Familias Unidas’ effects will be moderated by parental depression with the intervention being less effective in preventing/reducing depressive, anxiety symptoms; suicide ideation and behavior and drug use when parents have higher depressive symptoms.

We will also conduct moderation analyses to explore whether intervention impact on mental health and externalizing behaviors, including drug use, as well as sustainment, varies by quality of implementation at the levels of clinic and clinician. We will construct a clinic level measure of each implementation construct; for example, the Organizational Change Recipients’ Beliefs Scale [26], which measures acceptability, appropriateness, and feasibility of delivering eHealth Familias Unidas Mental Health, by clinic leaders, clinicians, office staff, and facilitators. We will construct an overall latent variable at each time that weights reporters’ scores by their role and accounts for number in that role in its measurement error. We will use the baseline latent score to test for variation in clinics’ effectiveness on 6-month mental health outcomes by implementation strength. Likewise, we will use the 6-month latent variable as a second- level mediator of impact on 18-month outcomes. We will also use the sustainment measure survey [33], collected in each clinic at 6 months of delivering eHealth Familias Unidas Mental Health, to predict sustainment of this intervention in the third phase of the study. The primary measurement of sustainment will be the number of families the clinic continues to enroll and deliver the intervention to after the formal yearlong implementation period has ended.

*Power analyses*. For continuous effectiveness outcomes, such as the slope of depressive symptoms over the 18 months, we have 87% power to detect an effect size of 0.5 (Type I error 0.05), which is the median effect size we found in a previous synthesis project investigating reduced depressive symptoms in 19 prevention programs with a two year follow-up [64]. We expect this effect size given that we are now including youth who are elevated on depressive or anxiety symptoms or have a history of suicide ideation or attempts. For suicide ideation, our growth models for repeated binary measures indicates we will have over 70% power to detect a yearly odds ratio (OR) reduction of 0.50 when the prevalence of ideation at baseline is 0.12 (or 12%). For suicide attempts, the baseline prevalence we have observed previously is 0.086 (or 8.6%), and this provides 55% power to detect an OR of 0.5. These ORs and prevalence rates were taken from our most recent Familias Unidas trials using the subset of youth with poor family communication, matching our inclusion criteria [18]. For suicide attempts, if we obtain an OR of 0.4, we will have 71% power to detect this change from baseline for attempts and 88% power for ideation. We will analyze impact on K-CAT MDD as well as use missing data and a two-stage design to calibrate these findings against K-SADS MDD. Thus, we have sufficient power to detect a large effect on suicide outcomes over time. For the power of detecting a mediation effect, we take as an example 6-month family communication mediating 18-month depressive symptoms. If both the a and b estimators have 80% power of rejecting their nulls, then the product has a 64% power of rejecting the mediation pathway as being significant. If each a and b estimators have 85% power, the product has 72% power of rejection.

Our primary approach to handling missing data will be to use Full Information Maximum Likelihood (FIML), which is implemented in M*plus* for multilevel modeling [61].

*Post-hoc analyses*. Several post-hoc analyses will be tested. First, we will examine condition effects on past 90-day frequency of alcohol and cigarette use. These outcomes are in line with the U.S. Preventive Services Task Force, which makes recommendations about preventive services to integrate within primary care. Impact on these outcomes would position eHealth Familias Unidas Mental Health as an intervention which should be integrated into primary care settings. Such a policy change would greatly enhance the sustainment of eHealth Familias Unidas Mental Health because an A or B rating would provide reimbursement for Medicaid and private insurance. We also recognize that there may be subgroups of Hispanic youth, such as LGBTQ, US born and acculturated Hispanic youth, girls, youth who experience ethnic bullying, families with high acculturative stress and low SES families who may be at higher risk for internalizing symptoms and suicide. As such, we will analyze youth differences in outcomes by these high-risk subgroups as well as difference in the mediation pathways towards reduced mental health problems and suicide related behavior and ideation.

## Discussion

In this manuscript, we described the rationale, design, and methods of an effectiveness-implementation hybrid type I intervention trial that will test the effectiveness of an eHealth intervention, *eHealth Familias UnidasMental Health*, in preventing/reducing depression symptoms, anxiety symptoms, suicide ideation and behaviors, and drug use among Hispanic adolescents. eHealth Familias Unidas Mental Health is a second online adaptation of the rigorously evaluated Familias Unidas intervention which has been found to prevent/reduce problem behaviors among adolescents across multiple iterations of the intervention including drug use [19, 20, 68, 69], sexual risk behaviors [19, 70, 71], externalizing behavior problems [11], internalizing problems [11, 17], suicide behaviors [18] and screen-based sedentary behaviors [72].

Considering the prevalence of mental health concerns among adolescents in general, and Hispanic youth in particular, scaling up evidence-based preventive interventions is crucial for the health of youth. However, despite the fact that 26.9% of 12–17-year-olds exhibited one or more mental, emotional, developmental, or behavior problems [73], implementation and sustainment of evidence-based preventive mental health interventions in community settings has been, and continues to be, a decades long challenge for the field of public health [74, 75]. Frequently cited, is the disconcerting reality that it takes approximately 17 years to implement merely 14% of evidence-based research outcomes in the real world [74, 76, 77]. The lack of access to quality mental health services is even more pronounced among racial and ethnic populations such as Hispanics [78] which is problematic because the health of the Hispanic population, whose population growth between 2010 and 2020 accounted for 51% of the total U.S. population growth [79], reflects the U.S.’s health. The rationale and methods of the current study have the potential to impact the implementation and sustainment of preventive interventions for mental health symptoms and drug use within primary care settings.

Although it is logical that preventive interventions backed by evidence should be available to youth, ensuring the adoption and sustainment of evidence based behavioral interventions within clinical settings is still important [74, 80]. A recent systematic review on sustainability strategies found 26 published articles that met the study inclusion criteria (public health evidence-based interventions, completed in a community or community-based setting, and included EBPI sustainment outcomes) and found a lack of consensus regarding a conceptual definition of sustainment, limited specification in terms of type of sustainment strategies, and a lack of application of a sustainment framework when reporting study activities [72]. These findings highlight that there remains a lot to be learned regarding sustainment of interventions in primary care settings. The data on organizational context and climate collected in this study will help identify barriers and facilitators for implementation, in addition to illuminating potential predictors of intervention sustainability. These data will inform what occurs after support from intervention developers and/or research staff has ended and whether the intervention is sustained.

Further, few preventive interventions have been evaluated for effectiveness among Hispanics in settings with high potential for implementation, such as primary care, in conjunction with an internet delivered modality. eHealth interventions such as the one being implemented in this study help address some of the barriers associated with maintaining adequate fidelity in community-based organizations in addition to the need for lower resources. For example, eHealth Familias Unidas Mental Health does not require facilitators for delivery of the parent or adolescent video sessions, therefore reducing burden on clinic staff which can potentially facilitate implementation and increase flexibility for participants.

This study, if successful, will provide evidence that eHealth Familias Unidas Mental Health can effectively reduce depressive and anxiety symptoms and prevent/reduce suicide attempts and ideation. The National Academy of Medicine and the National Research Council’s (2014) Forum on Promoting Children’s Cognitive, Affective and Behavior Health has indicated that rigorous evidence is necessary for the U.S. Preventive Services Task Force to make recommendations on integrating family-based prevention programs into primary care [81]. This study contributes to this much needed evidence and consequently has the potential to transform the way prevention programs are delivered in primary care.

## Supporting information

S1 Checklist SPIRIT 2013 checklist: Recommended items to address in a clinical trial protocol and related documents*. (DOC)

S1 File (PDF)

S2 File (PDF)

S3 File (PDF)
