# Supplementary material for: eHealth Familias Unidas Mental Health: Protocol for an effectiveness-implementation hybrid Type 1 trial to scale a mental health preventive intervention for Hispanic youth in primary care settings
Source: PLoS One. 2023 Apr 18;18(4):e0283987. doi: 10.1371/journal.pone.0283987 (PMC10112791; doi:10.1371/journal.pone.0283987)
Supplement: S1 File — (PDF) [file pone.0283987.s002.pdf]

1) Protocol Title

Scaling a Parenting EBI for Latinx Youth Mental Health in Primary Care

2) Objectives*

The goal of the proposed application is to evaluate in an effectiveness-implementation type I hybrid trial, an enhanced version of eHealth Familias Unidas for reducing depressive, anxious symptoms and suicide behavior in Hispanic youth. Using a randomized rollout design of 18 clinics, the study's aims are to:

AIM 1: Evaluate the effectiveness of eHealth Familias Unidas for Mental Health in preventing/reducing depressive, anxiety symptoms; suicide ideation and behavior; and drug misuse among those with poor family communication, elevated levels of depressive or anxiety symptoms, or a history of suicide ideation or behavior.

H1: eHealth Familias Unidas for Mental Health will be effective, relative to standard of care, in reducing these outcomes in Hispanic adolescents over time.

AIM 2: Determine whether intervention effects are partially mediated by family communication and externalizing behaviors, including drug misuse, and moderated by parental depression.

H2: Intervention effects on mental health outcomes will be partially mediated by improvements in family communication, externalizing behaviors (including drug misuse) and moderated by parental depression.

Exploratory AIM 3: Intervention impact on mental health and drug misuse as well as sustainment of the intervention in clinics will vary by quality of implementation at clinic and clinician levels.

3) Background*

Mental health problems among adolescents, including depression, anxiety, suicidal thoughts and behaviors, as well as drug misuse are a significant public health concern. National surveillance research indicates that approximately 14% of adolescents ages 12 to 17 reported having a major depressive episode (MDE) within the past year.[4] The death rate related to suicide, the second leading cause of death among adolescents in the United States (U.S.), increased 56% between 2007 and 2016, from 3.9% to 6.1%.[27] Among Hispanic adolescents, approximately 15% reported having a past year MDE in 2018, a significant increase compared to 12.6% in 2015.[28] Hispanic high school students reported higher rates of suicidal behaviors in the past year when compared to the general population of U.S. high school students, including having serious suicidal thoughts (16.7% vs. 15.8%) and suicide attempts (10.2% vs. 7.8%).[29] In particular, 21% of Hispanic females report suicidal thoughts when compared to 18.4% of non-Hispanic white females.[29] Further, mental health disorders developed during adolescence are shown to be associated with later negative health outcomes, including reoccurrence of depression and/or anxiety in adulthood,[30,31] anxiety disorders, bipolar disorder,[32] migraine headaches, poor self-rated health, low levels of social support,[31] suicide attempts, alcohol/drug dependence, life dissatisfaction, and risky sexual behaviors.[33]

Mental health disorders are comorbid with substance use behaviors among adolescents. Approximately 1.5% (358,000) of adolescents with an MDE also reported having a co-occurring substance use disorder (SUD).[4] Substance use behaviors, including illicit drug, marijuana, opioid, risky alcohol, and cigarette use, were more prevalent among adolescents who reported a past year

MDE than their adolescent counterparts without a past year MDE.[4] One study showed that 19.6% of Hispanic adolescents who reported suicide ideation also reported lifetime alcohol use, while 7.3% who reported suicide ideation did not report lifetime alcohol use.[22] Similar disproportionate findings were found for Hispanic adolescents who reported lifetime and past 3-month illicit drug use, with 20.8% reporting suicide ideation and 12.5% reporting suicide attempts, compared to 8.4% and 5.2% of adolescents who did not report lifetime and past 3 month illicit drug use, respectively.[22]

Evidence suggests family communication may work to reduce and/or prevent poor mental health and substance use outcomes in adolescents. For example, positive family relations were negatively associated with suicide risk among adolescents.[34] Additionally, parent-adolescent communication has been shown to be negatively associated with both internalizing and externalizing behaviors in adolescents.[35] Alternatively, negative family functioning, such as poor family communication, are shown to have adverse effects on adolescents' mental health.[36,37] It is possible that for certain youth, family functioning protects against poor mental health outcomes indirectly by reducing externalizing behaviors, including substance use behaviors. In fact, research indicates that Hispanic adolescents who reported increased levels of parent-adolescent communication concurrently reported lower levels of externalizing behaviors (i.e., conduct problems).[38] In another study, adolescent perceived parent communication was negatively correlated with both concurrent and subsequent past 30-day substance use behaviors.[39] The role the family plays on mitigating adolescent mental health disorders may be particularly applicable to Hispanic families due to cultural values related to commitment, loyalty, and obligation felt towards the family system.[40] Therefore, improving family communication should be an important focus when targeting the prevention of mental health and externalizing behaviors, including substance use, in Hispanic adolescents.

It is also important to take into consideration the effect of predisposing factors, such as parental depression, on adolescent internalizing behaviors. Reports show that 1 in 10 children in the U.S. have mothers who have experienced major depression in the past 12 months.[41] Adolescents whose parents are depressed are at greater risk for reporting poor mental health outcomes as well. In a parent-child dyad study with children between the ages of 7 and 17, children of depressed parents reported higher levels of depression when compared to children of non-depressed parents (7.87 versus 4.82).[42] Further, in a cognitive-behavioral preventative intervention targeted towards lowering adolescent depressive disorders, parental depression moderated intervention effects.[43] Specifically, when compared to usual care, the intervention resulted in lower rates of depression but only for adolescents whose parents were not depressed.

Considering the co-morbidity between mental health disorders and drug use behaviors and the association of family communication with both, it is not surprising that family-based interventions developed to address drug abuse prevention have also shown crossover effects in reducing the prevalence of mental health symptoms among adolescents. One example besides Familias Unidas is the Family Check-Up (FCU) intervention, which was designed to reduce adolescent substance use and behavior problems through improvements in parenting and family functioning.[44] Findings from the FCU intervention indicated that adolescents who engaged in the intervention reported lower levels of suicide risk in later adolescence and early adulthood.[34] Another example of a family-based intervention is the Family Bereavement Program (FBP) designed for children who experienced the loss of a parent, with participants showing decreased suicidal risk 15 years after participating in the intervention.[45] Our own synthesis of 19 adolescent preventive trials with a minority of these trials focused specifically on depression demonstrated a significant impact on depressive symptoms for two full years.[46]

Very few interventions have been culturally tailored to the needs of Hispanic families. One of these interventions is Familias Unidas.[16,47] Developed to address adolescent substance use and sexual risk behaviors by targeting family functioning, including family communication, Familias Unidas has also shown crossover effects lowering internalizing symptoms when compared to a control condition.[48] The intervention's effect on reduced levels of internalizing symptoms was partially explained by a moderated mediation effect of parent-adolescent communication. That is, there were stronger mediated effects for adolescents with lower levels of baseline levels of parent-adolescent communication when compared to adolescents who reported higher levels of parent-adolescent communication at baseline.[48] Additionally, among youth with externalizing symptoms, Familias Unidas had main effects on internalizing symptoms, one with moderated mediation on family communication[20] and one through cascading effects by improving family communication, which in turn reduced externalizing behaviors, which in turn decreased internalizing symptoms.[21] Finally, Familias Unidas significantly reduced suicidal attempts among adolescents with low levels of parent-adolescent communication.[22] Specifically, the intervention, relative to prevention as usual, reduced the risk of suicidal attempts for adolescents who reported poor parent-adolescent communication (see preliminary findings).[22]

Although intervention findings show promise, many of these interventions have not been assessed outside of a research setting, and thus, a knowledge gap exists on the successful integration of mental health evidence-based interventions into delivery systems such as health care systems. Primary care settings may afford an opportunity for successful implementation of evidence-based preventive interventions for three main reasons. First, primary care settings are visited often by adolescents. In 2017, approximately 74% of Hispanic adolescents under the age of 18 reported seeing a doctor or another health care professional in the last 6 months.[49] Second, primary care doctors are seen as a trusted source of health information, such that parents often consult primary care providers with concerns they have regarding adolescent behaviors.[50]Third, primary care settings may provide a solution for the underutilization of mental health care services by de-stigmatizing access to care. Hispanic populations are less likely to seek or use mental health services when compared to non-Hispanic white populations, and they rely on informal sources of support and primary care providers for help with mental health problems.[51] For these reasons, existing research suggest that preventative mental health services can and should be provided in primary care settings in an effort to reduce the mental health disparities seen in Hispanic populations.[52] Having primary care settings sustain interventions such as eHealth Familias Unidas after the research study ends is important. Such sustainment may vary as a function of clinic organization, organizational climate for evidence-based implementation, and clinic leadership (Aarons et al., 2015, 2017).

Despite the possible benefits of implementing evidence-based interventions in primary care settings, challenges exist. Commonly reported barriers by primary care personnel when implementing an intervention are time constraints and existing workloads.[53,54] A possible solution to the limited time and resources afforded in primary care settings could be relying on internet-based (i.e., eHealth) interventions. According to the Pew Research Center, at least 80% of Hispanic adults access the internet via a mobile device such as a cellular phone or tablet).[55] Further, trends indicate an increase among Hispanic adults in accessing the Internet via mobile devices, specifically from 76% in 2012 to 94% in 2015 (compared to 85% in non-Hispanic Whites), among internet users. Scientifically based and appropriately designed eHealth interventions[57] offer the advantage of also reaching at-risk Hispanic adolescents while cutting the cost of relying on mental health providers.[58] The feasibility and acceptability of an eHealth version of Familias Unidas has previously been examined, with high intervention engagement rates (84%) and positive participant feedback on the content of the intervention.[59] Further,

eHealth Familias Unidas was found to be efficacious in reducing drug use, prescription drug use, and cigarettes use trajectories when compared to prevention as usual and improving overall family communication at 3 months post-baseline.[60]

Preliminary studies.

Efficacy Study of Face-to-Face Familias Unidas in a School Setting. Prado and colleagues[18] evaluated the efficacy of Familias Unidas in preventing adolescent drug use, cigarette use, alcohol use, and unprotected sexual behavior, relative to (1) an HIV preventive intervention and (2) a cardiovascular preventive intervention. Participants (n = 266, mean age = 13.4, *SD* = .68) were assessed at baseline, randomized, and reassessed at 6, 12, 24, and 36 months post baseline. Familias Unidas was efficacious, relative to the cardiovascular intervention in reducing past 90-day illicit drug use ($z = 2.02$, $p < .05$), efficacious relative to both the HIV intervention ($z = 3.25$, $p < .002$) and the cardiovascular intervention ($z = 2.66$, $p < .008$) in reducing past 90-day cigarette use, and in reducing unprotected sexual behavior at last sexual intercourse ($\chi^2(1) = 3.87$, $p < .05$). The effects of Familias Unidas were mediated by improvements in family communication.

Replication efficacy study of Face-to-Face Familias Unidas in a School Setting. Prado and colleagues[17] evaluated the efficacy of Familias Unidas in reducing past-90 day illicit drug use and unprotected sexual behavior, relative to prevention as usual, among adjudicated Hispanic youth. 212 (mean age = 14.6, *SD* = 1.36) juveniles and their primary caregivers were assessed at baseline and reassessed at 6 and 12 months post baseline. The results showed that Familias Unidas was efficacious, relative to prevention as usual, in improving family communication ($b=.28$, $p=0.02$), increasing past 90-day condom use ($b=0.68$, $OR = 1.97$, $p=0.015$), and in reducing past 90-day illicit drug use ($b=-0.72$, $p=0.04$).

Effectiveness Study of Face-to-Face Familias Unidas in a School Setting. Prado, Estrada and colleagues[66] evaluated the effectiveness of face-to-face Familias Unidas in reducing the frequency of drug use, alcohol use, and unprotected sexual behavior, relative to prevention as usual, among 746 Hispanic youth (mean age = 13.9, *SD* = 0.67) recruited from 18 middle schools. School counselors were trained to deliver Familias Unidas. Participants were assessed at baseline and reassessed at 6, 18 and 30 months post baseline. The results showed that Familias Unidas was efficacious, relative to prevention as usual, in increasing family communication ($b=-0.148$, $p=0.014$), reducing the frequency of past 90-day illicit drug use ($b=0.23$, $p<0.001$), and in reducing past 90-day unprotected sexual behavior ($b=0.093$, $p=0.008$).

Efficacy Study of eHealth Familias Unidas in School Settings. Estrada, Prado and colleagues[67] evaluated the efficacy of an eHealth adaptation of Familias Unidas in reducing substance use and condomless sex among 230 Hispanic youth (13.6 years, SD = 0.7). Youth were assessed at baseline, 3 and 12 months post baseline. Significant intervention effects were found across time for drug use ($b = -1.16$, $p< .01$), prescription drug use ($b = -1.16$, $p< .01$), and cigarette use ($b = -1.05$, $p< .01$). Effects were not found on condomless sex. In our past trials, significant effects on condomless sex began to emerge at 24-month post baseline.

Pilot Study and Ongoing Study of eHealth Familias Unidas in Primary Care. Prado and colleagues[25] evaluated and established the feasibility and acceptability of delivering eHealth Familias Unidas in a universal sample of 33 Hispanic youth recruited from three primary care settings. This study led to the next trial.

Relative Effectiveness of eHealth Familias Unidas in Primary Care to Prevent Drug Misuse and STIs. This ongoing NIDA funded study uses a sample of 456 Hispanic youth recruited from 16 diverse primary care clinics. Only baseline, 6-month, and 12-month post-baseline data collection have been completed, and eHealth Familias Unidas has already shown an effect on

family functioning, a mechanism of action of the intervention. The percentage of sessions attended by the families randomized to the intervention exceeded 70%. Over 85% of families have currently been retained in the follow-ups.

Crossover Effects on Internalizing Symptoms and Suicide Ideation. Although eHealth Familias Unidas does not directly address internalizing symptoms, Familias Unidas has had crossover effects on both internalizing symptoms (consisting of depression and anxiety symptoms) and suicide ideation. One study led by Perrino and colleagues[48] found that, relative to control, Familias Unidas reduced internalizing symptoms among 242 delinquent 13- to 17-year-old youth. Improvements in family communication mediated the intervention's effects on internalizing symptoms for youth whose families had poor communication. A second study of 721 Hispanic youth (14.0 years; SD=1.14) found reductions in internalizing symptoms for youth with low family communication through intervention-mediated improvements in communication across three pooled Familias Unidas trials.[20] A third study among youth with externalizing behaviors documented cascading effects on internalizing symptoms through the intervention's impact on improving family communication, followed by subsequent reductions in externalizing behaviors, and ultimately reduced internalizing symptoms.[21] In terms of suicide, a fourth study of a single trial showed that at baseline 9.2% of adolescents had recent suicide thoughts and 5.7% a suicide attempt,[22] with 12.2% reporting thoughts and 8.6% reporting attempts among the 18.8% of those reporting poor family communication. This study found that Familias Unidas significantly reduced suicide attempts compared to control ($OR = .53$, $p < .05$) for youth reporting poor family communication.

It is important to note that the effects sizes between eHealth Familias Unidas and Control vs Face-to-Face Familias Unidas and Control in prior studies have not been significantly different across the various outcomes (except for smoking which is not an outcome in this study). Additionally, participants in eHealth Familias Unidas had greater attendance than those in the face-to-face version of the intervention,[68] and attendance in the invention is related to improved study outcomes.[69]

Although the original Familias Unidas and eHealth Familias Unidas interventions do not specifically target youth internalizing symptoms, studies have demonstrated cross-over effects on mental health, reducing youth internalizing symptoms and suicide attempts[20,22,48]. These crossover effects may be explained by common underlying parent and family risk and protective factors associated with adolescent depressive, anxiety, and suicide symptoms. Common risk factors include family conflict and parental rejection, while common protective factors include positive family communication and parental warmth.[70-72] Positive parenting and family functioning are also protective for adolescent externalizing symptoms and drug use,[73] which are risk factors for later internalizing symptoms.[21] It is therefore not surprising that Familias Unidas has been found to reduce multiple youth mental health and behavioral outcomes through intervention-mediated improvements in family factors, such as family communication.[21,48] Other preventive interventions targeting parent and family factors have documented improvements in internalizing symptoms through their impact on these common intervention mechanisms, such as by reducing family conflict or improving parent-child.[74,75] Additionally, poor mental health in parents can interfere with the effects of programs that aim to prevent adolescent mental health problems.[76] As a result, addressing parent mental health is an important objective in programs to promote adolescent mental well-being.

4) Inclusion and Exclusion Criteria*

Families will be screened based on the following inclusion criteria:

(a) Female and male adolescents, who self-identify as Hispanic (or Latino(a))

(b) Adolescent between the ages of 12 – 16 years
(c) Adolescent living with an adult primary caregiver who is willing to participate
(d) Families must have broadband internet access on a device, including (but not limited to) a smartphone, iPad, tablet, computer at their home or other location (e.g., school, library, etc.)
(e) Families screening positive on poor family communication (a score of less than 75 on the communication measure; see measures) or youth reporting elevated depressive or anxiety symptom scores or a history of suicide behavior (ideation or attempts)

Exclusion criteria: Families reporting plans to move out of the South Florida area during the study period.

5) Procedures Involved*

Design. To evaluate eHealth Familias Unidas Mental Health in an effectiveness-implementation hybrid Type 1 trial, this study will use a rollout design, which is an extension of a Stepped Wedge design. We will balance, and then randomize all 18 sites into five cohorts of 4 or 3 clinics to when they would end their control phase, when they would begin receiving eHealth Familias Unidas Mental Health, and when they would start the sustainability period where UM staff would no longer provide formal support (i.e., clinical supervision) to the clinic to deliver eHealth Familias Unidas Mental Health. This design is an extension of a Stepped Wedge design since it has three conditions (as shown in the figure) and provides both within and between clinic comparisons of effectiveness over time. Youth and parents will be assessed at baseline, 3-, 6-, and 18-months post-baseline. We will collect electronic health record data (e.g., exposure to an antidepressant) for participants during their control phase.

Participants. The sample will consist of 468 Hispanic female and male adolescents and their primary caregivers recruited from one of 18 primary care clinics, who meet the inclusion/exclusion criteria below. We will include a mix of naive and previously involved clinics in the proposed study. Because we did not attempt to institutionalize eHealth Familias Unidas in earlier involved clinics, we do not anticipate much difference in effect, but we will test for this.

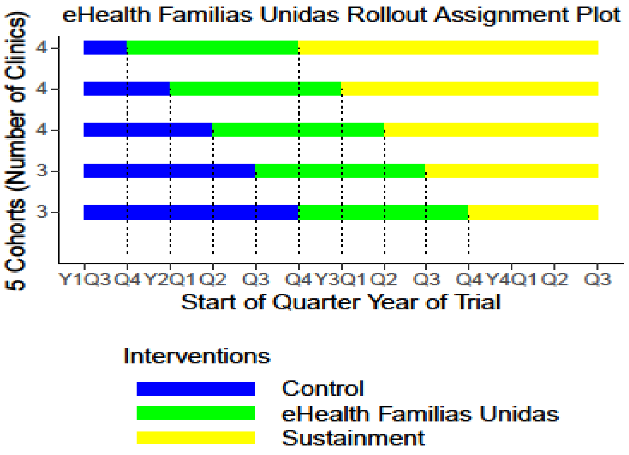

A document to assess for the inclusion/exclusion criteria will be included as part of the patient check-in process. We will screen parents and adolescents separately to verify the inclusion and exclusion criteria. Adolescents will be asked questions regarding their mental health (reporting elevated depressive or anxiety symptom scores or a history of suicide behavior) and parents will be asked questions related to communication with their adolescent.

Recruitment. Participants will be recruited from 18 primary care clinics.

The sites are heterogeneous and consist of federally qualified health centers, academic primary care clinics located within the University of Miami's Health District, and private community clinics. Collectively, these sites jointly serve over 10,000 Hispanic between the ages of 12 -16 per year. Therefore, we do not anticipate any challenges in recruiting 468 youth over the 18-month recruitment period.

Families who meet the study's inclusion/exclusion criteria will automatically be connected via protected videoconference software to the research team via an iPad. The team member will explain the study to eligible participants and complete the e-consent process. Families who enroll will then be sent a link to complete the assessment battery on the iPad while they wait to see the physician. The clinic staff member will thank the study participant for enrolling in the study and will make a hand off to the physician. If the family is unable to complete the survey at the clinic, a link will be sent to them so that the survey can be completed at their convenience. Families will also have the option to complete the follow-up surveys via a link that is emailed to them.

As an additional form of recruitment, clinic doctors will also provide families with a note where they recommend the program. If the family is interested in participating, they will provide their contact information and a research team member will call them to explain the study.

Intervention conditions

eHealth Familias Unidas Mental Health. The experimental condition consists of *eHealth Familias Unidas Mental Health*, an enhancement of the eHealth Familias Unidas intervention.[79] eHealth Familias Unidas Mental Health's targets mental health and suicide ideation and behavior. The intervention is based on ecodevelopmental theory,[80,81] a contextual, multi-level framework that provides a useful way to organize and address risk and protective factors for youth drug use, externalizing behaviors, sexual risk behaviors and internalizing symptoms from the macrosystem (i.e., the broad societal factors and philosophical ideals that define a particular culture - e.g., Hispanic cultural values and norms) to the microsystem (i.e., the contexts in which the adolescent participates directly - e.g., family, peers). This theory helps frame prevention intervention efforts by addressing the multiple risk and protective factors operating at different levels of adolescents' social contexts that influence adolescent health. As originally developed, eHealth Familias Unidas consists of 12 sessions: eight (20 – 40 minute) video parent group sessions in Spanish (with English subtitles) and four (45-minute) web-conferenced family sessions delivered in either Spanish and/or English, depending on the parent level of comfort with either language. In past trials, 99% of parents understood Spanish, including those who preferred or were more comfortable with English).

eHealth Familias Unidas Mental Health builds upon the original, evidence-based eHealth Familias Unidas intervention,[62,79] adding components that more directly address internalizing symptoms and suicide behavior. Specifically, the adapted intervention adds two online video sessions, one each for parent and adolescent, which focuses on promoting mental health by strengthening family and interpersonal protective factors. The sessions educate youth and parents about mental health symptoms and suicide and provide a screening tool for those reporting high symptom levels of distress. The team's licensed mental health provider, Ms. Tapia, will refer to care in the community, as appropriate. The new sessions build heavily upon the existing family communication module to highlight how this proven protective factor provides opportunities for listening and monitoring youth mental health. Family communication is a teachable skill and modifiable protective factor that can reduce adolescent isolation and enhance social support, as well as provide opportunities for warm and positive parent-adolescent relations that are marked by less conflict. Importantly, the parent session addresses parent self-care, how parent mental health affects family functioning, including family communication, parenting and ultimately

adolescent well-being. These added components are culturally consonant for Hispanic families, allowing reinforcement of family interconnectedness and the role of parents as competent family leaders. These values are congruent with the core aspects of Familias Unidas, including mechanisms of action, such as promoting family communication. Mental health is weaved into several of the existing intervention sessions. For instance, in the "parental monitoring of peers" session, the module addresses developmentally appropriate ways for parents to monitor and help strengthen positive adolescent peer relationships to address internalizing symptoms, e.g., managing peer rejection, bullying, and other peer-related mental health risks. eHealth Familias Unidas Mental Health maintains its original focus on drug use and sexual risk, given the high comorbidity that these have with internalizing symptoms and suicide risk. For instance, the drug use module integrates the role of drugs in exacerbating internalizing and suicide symptoms, as well as the role of internalizing symptoms in fueling drug use. Only four of the 13 sessions will be delivered live online (similar to telemedicine) via a clinician. In our most recent trial,[79] 72% of families participated in all intervention components (both completed all live sessions and watchedall the videos).

Video parent group sessions consist of: 1) a culturally syntonic telenovela/soap opera series, 2) videotaped parent group discussions, and 3) interactive exercises. Sessions are viewed online by each parent.

Telenovela/Soap opera. The telenovela highlights Hispanic adolescents' risk for drug use and sexual risk behavior and were produced in ways that are culturally syntonic and consistent with the intervention's theoretical framework (e.g., placing parents in the leadership role in their families). The telenovela consists of eight episodes that include scenes aimed at facilitating process conversations, improving parents' understanding of the risks of drug use and sexual risk behaviors, and the ways to respond to opportunities that youth face for drug use, sexual risk behaviors, and internalizing and suicide symptoms. Each episode lasts approximately five minutes and provides a scene that is directly related to the content covered in each of the nine parent video group sessions. A telenovela session focused on the prevention of youth internalizing symptoms and suicide will be developed, reviewed by our expert advisors, and produced for this enhanced mental health intervention. The additional telenovela episode will be developed in a similar manner as done with previous episodes. In fact, this process has already begun. A draft of the episode script was developed that is culturally syntonic and reflective of the topics of depression, anxiety, and suicidality. The script was developed with feedback from co-Investigators, and 15 parent-youth dyads. If the study is funded, we will also request feedback on the draft from our Scientific Advisory Board before it is produced by graduate students at the University of Miami's School of Communication. We have also developed draft prototypes for the other new intervention modules (e.g., youth online session).

Parent Video Group Discussions. The parent video group discussions of eHealth Familias Unidas were developed as pre-recorded sessions (eight total), and we will add a ninth to cover internalizing symptom and suicide prevention; they simulate the face-to-face group experiences in earlier deliveries of Familias Unidas. Participants in these enactments are parents from the community with an adolescent between the ages of 12 –16. Parents were invited to participate in the videotaped Familias Unidas parent group discussions/sessions, which were led by Ms. Tapia, the Familias Unidas clinical trainer and supervisor. These sessions were video- recorded and edited so that they succinctly delivered the important core intervention components of the face-to-face Familias Unidas intervention.

Interactive exercises. Interactive exercises are used to reinforce session content and to incorporate the participatory learning strategy used in the face-to-face intervention. These interactive exercises are embedded in the parent video group sessions. The type of interactive exercise varies from session to session and includes true/false questions, multiple-choice questions, and point-

and-click responses. Parents are provided with instant feedback regarding their responses and all on-screen text (e.g., instructions, response choices, answer summaries) is paired with audio to reduce literacy-related barriers. The intervention facilitators are able to review parents' responses and use this information to facilitate discussion between parents and adolescents during the online family sessions.
Youth online session. One online module for youth will be developed to address interpersonal and social protective factors for youth internalizing and suicide symptoms, including family and peer relations and skills. The video session will be delivered online to each youth. The video session will involve psychoeducation regarding youth mental health symptoms and risks, and completion of a self-screening tool for these symptoms. Youth showing clinically-relevant symptoms will be contacted by the team's licensed mental health provider, Ms. Tapia, for referral to care in the community. This module addresses youth coping and interpersonal skills, along with interactive exercises including multiple-choice questions, and point-and-click responses. Although this is the one single youth only session that addresses protective factors for internalizing and suicidal symptoms, it is important to note that other, existing sessions of the intervention focus on well-supported interpersonal and family protective factors for internalizing symptoms and suicide behaviors (e.g., family relationships and support, drug misuse).

Online Family Sessions. The four (45 minute) online family sessions are delivered live via the internet utilizing webcams and Zoom videoconferencing software. eHealth Familias Unidas Mental Health facilitators will be responsible for scheduling and delivering the four online family sessions with each of their families. The facilitator and one family (i.e., adolescent and parent) participate in each of the family sessions which will provide parents the opportunity to practice with their adolescent the skills that they learned in the parent video group sessions.

The four virtual family sessions will be conducted by a trained study team member or pediatric staff member (elsewhere, "Facilitator") according to the same protocol used in the face-to-face Familias Unidas intervention. However, eHealth Familias Unidas Mental Health will use online video conferencing technologies to virtually recreate the therapeutic environment of the family session. The online family sessions will provide parents the opportunity to practice with their adolescent the skills that they learned in the video parent group sessions. The content of each of these four family sessions is identical to that of face-to-face Familias Unidas. Virtual family sessions will be conducted in Spanish and/or English to accommodate the language preferences of the parent and adolescent participants of each family session. Pending parent and adolescent consent and assent, all virtual family sessions will be video-recorded via Zoom. All recorded sessions will be stored in secure University of Miami servers and will be labeled with the family's case ID. The recordings will only be used for purposes of clinical supervision, research and training—provided participant consent/assent.

Other family members (adults or minors, e.g., sibling at least 12 years old, grandparent, other guardian) can participate in the virtual family sessions. The only requirement is to obtain their secondary consent. (1) For other adults, a **secondary adult consent** must be signed to accept participation in the study. (2) For other minors, the staff needs to get signed by one parent or legal guardian, the **secondary adult consent for a minor**, and the **secondary minor assent** signed by the minor. These secondary participants will not participate in the assessment battery or other intervention activities unless it is a second parent (i.e., legal guardian) in which case this person would sign the main consent form.

A log-in procedure accessed through the eHealth Familias Unidas website will serve as the

mechanism by which to ensure secure access and confidentiality for participants. Each participant will be assigned a unique log-in name and password, which will provide them access to the eHealth Familias Unidas Mental Health session. The log-in procedure will also facilitate the close monitoring of participants, particularly as it relates to session participation rates. Specifically, the log-in procedure will be utilized to record who (i.e., which participant), when (i.e., day/time), how long (e.g., time of session attendance), and for what purpose (e.g., which session) the intervention website was accessed.

Our information technology team employs the latest technology to ensure security and confidentiality of participants, including but not limited to data error checking through forms, filtering output, and the use of encryption. Server Port Hardening will be implemented to keep the web server safe from intrusion. Additionally, a Secure Socket Layer Encryption certificate will be issued to help prevent Internet fraud. The implementation of SSLs (i.e., cryptographic protocols) aims to enhance security over the Internet. IT will conduct stringent testing of web applications prior to the implementation of eHealth Familias Unidas Mental Health to prevent any exploits from occurring and to ensure the confidentiality of participants.

-All participants will not receive the intervention. Participants will receive the intervention depending on when the clinics offer the program, which is based on the study's randomization. After the intervention is complete, assessors will call the families to return to complete follow upassessments. The follow up assessments will occur online through links emailed via REDCap.

The follow up assessments collect the same data as the baseline assessment. All measures will be self-report. We will ask participants to give us the names and telephone numbers of three people who will always know how to reach them, in case we lose contact. We will never share anything participants tell us with these people.

Measures.

Below we list the measures that will be administered to participants:

Demographics. A demographic form developed by the creators of Familias Unidas will be administered to caregivers and youth in order to collect information on contact information, age, date/place of birth, number of years residing in the U.S., parents' marital status, gender identity, and family income. Further, five questions have been created to assess participant understanding of the consent form. Sometimes people in our studies move and we can't find them. Therefore, we will ask parent participants to give us the names and telephone numbers of three people who will always know how to reach them, in case we lose contact and can not reach them for the follow-up assessments.

Family functioning (reported by both youth and parents) consists of parental investment in the adolescent and parent-adolescent communication. Parental investment in the adolescent will be assessed using 1) the Parenting Practices (Loeber et al., 1999), and 2) the Family Relations Scale (Tolan et al., 1997). Two subscales of the Parenting Practices:

(a) extent of parental involvement (20 items) and (b) positive parenting (9 items) will be used to assess parental investment. Cronbach's alpha for the two (parent reported) subscales were .83 and .78, respectively. Cronbach's alpha for the two (adolescent reported) subscales were .86 and .85, respectively. The Family Relations Scale assesses the amount of social support the adolescent receives from the parent (6 items) (Tolan et al., 1997). Cronbach's alpha for this subscale is .73 (parents) and .70 (youth).

Communication will be assessed via the Parent-Adolescent Communication Scale (Barnes & Olson, 1985), which assesses the quality and content of communication between parents and youth. Youth report on the quality and content of communication they perceive with their parent. In the parent version, parents report on their perception of the quality and content of their communication with their youth. Cronbach's alpha coefficients for this measure was .85 (parents) and .93 (youth).

Youth's own sexual risk behaviors will be assessed using a 22-item instrument from Jemmott, Jemmott, and Fong (1998). This self-report measure will be used to document the number of times youth report having sex (oral, vaginal, and anal) in their lifetime and in the past 3 months. This instrument is also used to document the number of times youth has been exposed to situations of high risk during sexual intercourse (e.g. having unprotected sex and/or being under the influence of alcohol or drugs while having sex). This measure also asks participants whether they have sex with males and/or females.

Youth's and parents' own drug use will be measured using items used as part of the Monitoring the Future survey (Johnston et al., 2010). This 47-item self-report instrument assesses lifetime drug use and the frequency of drug use (by type of drug) in the 3 months prior to assessment. Additionally, questions regarding the use of e-cigarettes and hookahsfor lifetime use, the previous 90 days and previous 30 days will be asked.

Finally, three questions regarding second hand smoking will be asked:

How much do you think you are exposed to tobacco smoke at home?" (select one: not at all, somewhat, moderately, a lot, extremely

How much do you think you are exposed to tobacco smoke outside of home?" (select one: not at all, somewhat, moderately, a lot, extremely

How many years in total did you live in the same household with someone else who smoked tobacco products?"

The Klein Sexual Orientation measure (adolescents only, 8 items) will be used to assess adolescent sexual orientation and preferences. Parents will also be asked the following questions regarding sexual orientation:

a.-Homosexuality should be:

Accepted by society

Discouraged by society

Refuse to answer

b- If your son or daughter discloses to you that she or he is sexually attractive to someone of the same sex, for example, your son is sexually attractive to another boy or your daughter to another girl. Will you support him or her?

YES NO MAYBE

c- If you find out that your son or daughter is sexually attractive to someone of the same sex. Will you be willing to enroll in a program with your child that will help you both during this process?

YES NO MAYBE

The CES-D (parents and adolescents, 20 items) will be used to assess symptoms of depression.

ACES (for parents only, 17 questions) will assess adverse events before the age of 18 for parents

The CASA (for parent and adolescent, 8 items) will assess use of community services.

The Revised Behavior Problem Checklist (RBPC; parent only, 55 items): the externalizing subscales of the RBPC will be used to assess adolescent problem behaviors from the parent perspective.

The externalizing subscales of the Youth Self Report (YSR; adolescent only, 32 items) will be used to evaluate adolescent problem behaviors from the adolescent perspective.

Health related quality of life (QOL; parent and adolescent, six items) will be used to examine physical, mental and emotional health.

CRAFFT (adolescent, 10 items): assesses the consequences of alcohol and drug use.

Given the opioid epidemic, the following questions will be asked of the adolescent assessment:

a. Have you ever used any pain killers (opioids) without a doctor's prescription?

b. Have you used any pain killers (opioids) in the last 90 days, without a doctor's prescription?

c. Have you used any pain killers (opioids) in the last 30 days, without a doctor's prescription?

d. Have used any pain killers (opioids) in the last seven days, without a doctor's prescription?

We would like to collect adolescents' email addresses in the event that we cannot reach them for the follow-up assessment and/or other study related information. The text will include the following:

We are requesting your email address in the event that we cannot reach you for the

follow-up assessments and/or other study related information. Please choose one of the following:

______Yes, you may use email to contact me for this study. My email address is: ______________________

______No, I do not want to be contacted by email.

Five questions have been created to assess participant understanding of the consent form. We would also like to collect data from clinic facilitators, clinic leaders, and physicians to assess organizational level factors and attitudes towards evidence-based interventions. These clinic facilitators, clinic leaders, and physicians will be paid $20 for completing this information, we will ask them to complete this measure three times: pre-intervention, post-intervention and during the sustainment period.To do this, we will use the below measures:

Attitudes Towards Evidence-Based Practice Scale (EBP; Clinical Directors, Clinicians, Office Managers, Facilitators; 15 items): this measure will be administered to facilitators to assess their views on evidence-basedpractices.

Multifactor Leadership Questionnaire (clinic leaders/physician, 21 items): this measure will be administered to clinic leaders/physicians to assess transformational and transactional leadership behavior.

Implementation Leadership Scale (ILS; Clinical Directors, Clinicians, Office Managers, Facilitators , 11 items): this measureassesses the degree to which organizational leaders support their staff in the implementation of evidence-based interventions

Implementation Climate Scale (ICS; clinic leaders/physician, 18 items): assesses the level of astrategic organizational climate within an organization.

Organizational Change Recipients' Beliefs Scale (OCRBS; Clinical Directors, Clinicians, Office Managers, Facilitators, 24 items): measures acceptability, appropriateness, and feasibility of delivering the intervention by different staff.

Sustainment Measurement System (SMS; Clinical Directors, Clinicians, Office Managers, Facilitators, 52 items): measures the sustainability of the intervention by assessing the number of families the clinic continues to enroll and deliver the intervention to after the implementation period has ended.

Additional Measures to include:

Brief Resilience Scale (BRS; parent and adolescents; 6 items)
Perceived Stress Scale (PSS; parent and adolescents; 4 items)
Multidimensional Perceived Support Scale (MSPSS; parent and adolescents; 12 items)
Bicultural Stress Scale (BSS; adolescent and parent; 20 items)
Perceived Discrimination Scale (PDS; adolescent only; 7 items)
CDC Bullying (BLY; adolescents only; 4 items)
Columbia Impairment Scale (CIS; adolescents only; 13 items)
Introspection (EDI; adolescents only; 10 items)
DSM5 Cross-Cutting Child (DSMCC; adolescent only; 25 items)
DSM5 Cross-Cutting (DSM; parent only; 23 items)
Generalized Anxiety Disorder (GAD; parent and adolescent; 7 items)
PHQ-9 (12yrs +) (PHQ; adolescents only; 9)
Revised Children's Anxiety and Depression Scale (RCADS; parent only; 25 items)

RCADS25 Youth (RCADS; adolescent only; 25 items)
Suicide Behaviors Questionnaire Revised (SBQR; adolescent only; 4 items)
Columbia Suicide Screening (CSSR; adolescent only; 6 items)
WHO Disability Assessment Schedule (DAS; parent only; 15 items)
Vancouver Index of Acculturation (VIA; parent and adolescent; 20 items)

Parenting Relationship with Peer Group (parent and adolescent; 5 items)

6) Data and Specimen Banking*

☒ This section is not applicable. This research is not banking data or specimens for future use.

7) Data Management*

Data Management & Quality Control Procedures. Assessment data will be entered directly by participants onto tablets via REDCap, which is linked to secure university servers. We will follow procedures to ensure quality control in the collection, verification, and documentation of data that we established in previous trials. Data files will be exported from REDCap, will be cleaned and reviewed, and prepared for analysis with analysis programs, including M*plus*.

Data Preparation & Preliminary Analyses. Testing of distributional assumptions will include statistical tests for univariate and multivariate normality (tests of skew & kurtosis) as well as visual inspections of the empirical distributions for the data at each time point. Should deviations be deemed sufficient for concern, transformation of variables will be attempted where possible. The distribution of our outcomes will be skewed, and methods specifically used for such data will be used. Often such data follow a Poisson distribution, and a Poisson regression[104,105] or a two part growth curve model[106] would be the appropriate way to handle these analyses in the event of positively skewed distributions. Reliability estimates of internal consistency (Cronbach's α) will be generated for all scale scores. If reliability estimates are found to be below .80, item total correlations and factor analyses will be employed to diagnose and correct psychometric problems.[107] To account for multiplicity adjustments in the proposed analyses below, we will sensitive analyses for primary outcomes.

AIM 1: Evaluate the effectiveness of eHealth Familias Unidas for Mental Health in preventing/reducing depressive, anxiety symptoms; suicide ideation and behavior; and drug misuse among those with poor family communication, elevated levels of depressive or anxiety symptoms, or a history of suicide ideation or behavior.

For continuous measures of depressive and anxious symptoms and drug misuse, we will analyze impact using growth modeling with four time points modeled as a multivariate normal distribution. For suicide behavior (i.e., ideation and attempt), and drug misuse, we will conduct generalized growth modeling with mixed effects using these repeated binary measures. For both continuous and binary outcomes, our analytical procedures will follow those we have used previously to analyze Familias Unidas trial data. For our standard analyses we will use Mplus[108] to examine changes in growth using its two-level modeling of clinic and individual, commonly called three-level modeling in the mixed effect modeling literature (individuals with repeated measures nested within clinics,[109,110]). Since youth are recruited from all 18 clinics both during Control and eHealth Familias Unidas Mental Health, we can consider clinic to be a "blocking

factor," so the overall effect is essentially a mean of the differences in response across all 18 clinics. After suitable transformation of the time axis, handling of missing data, and model fit checking (see Clinical Trials Section 4B for additional details), we will use a Wald type test on the difference in mean slope parameters for eHealth Familias Unidas versus Control assigned participants. Base rates for suicide attempts are low and thus we accounted for them in our analytic plan and power analyses (see below). In a universal trial of Familias Unidas, found that at baseline 9.2% of youth reported suicide ideation and 5.7%reported a suicide attempt in the preceding year.[22] We have presented power analyses below based on these low but not insignificant rates. Finally, to ensure that our final timepoint which has a longer time gap does not overly influence the findings, we will examine transformations of the time axis.[46]

AIM 2: Determine whether intervention effects are partially mediated by family communication and externalizing behaviors, including drug misuse, and moderated by parental depression.

For mediation analyses, we will use the "product of coefficient" approach[111] that we used in previous analyses to test for an indirect effect of improved family communication by 3 months, decreased externalizing behaviors by 6 months, and further decreased depressive and anxious symptoms as well as suicide ideation and attempts by 18 months. We analyze this in 2 parts, with family communication affecting externalizing followed by externalizing behaviors affecting depressive symptoms. For the second model, the product of the effect of family communication on intervention (a coefficient) and depression on family communication controlling for intervention (b coefficient) measures this latter indirect effect. We will use M*plus* to compute bootstrapped confidence intervals since this product has a very non-normal distribution. Similarly, we will examine whether externalizing behaviors (including drug misuse) change by 6 months mediates the 18-month depressive, anxious, and suicide behavior outcomes. In addition, we propose to test whether the slope of externalizing behaviors changes on 18-month outcomes as well. To examine moderation by parental depression, we will test for an interaction with that symptomatology at baseline and intervention, both in growth models and in 18-month outcomes. We hypothesize that Familias Unidas' effects will be moderated by parental depression with the intervention being less effective in preventing/reducing depressive, anxiety symptoms; suicide ideation and behavior and drug misuse when parents have higher depressive symptoms. Parental depression can significantly impair a person's ability to parent as well as their family functioning[112] and past prevention programs have found that parent depression moderates preventive intervention effects in this manner.[76]

Exploratory AIM 3: Intervention impact on mental health and externalizing behaviors, including drug misuse, as well as sustainment, will vary by quality of implementation at the levels of clinic and clinician.

For this aim, we will construct a clinic level measure of each implementation construct; for example, the Organizational Change Recipients' Beliefs Scale[97], which measures acceptability, appropriateness, and feasibility of delivering eHealth Familias Unidas Mental Health, by clinic leaders, clinicians, office staff, and facilitators. This measure will be collected immediately after training the facilitator is complete in each clinic, and then again 6 months after starting to deliver this intervention. We will construct an overall latent variable at each time that weights reporters' scores by their role and accounts for number in that role in its measurement error. We will use the baseline latent score to test for variation in clinics' effectiveness on 6-month mental health outcomes by implementation strength. Likewise, we will use the 6-month latent variable as a second-level mediator of impact on 18-month outcomes. We will also use the Sustainment Measurement System Survey, collected in each clinic at 6 months of delivering eHealth Familias Unidas Mental Health, to predict sustainment of this intervention in the third phase of the study. The primary measurement of sustainment will be the number of families the clinic continues to enroll and deliver eHealth Familias Unidas Mental Health after the formal yearlong

implementation period has ended (see Clinical Trial Section 4B for more details).

Power Analyses: For continuous effectiveness outcomes, such as the slope of depressive symptoms over the 18 months, we have 87% power to detect an effect size of 0.5 (Type I error 0.05), which is the median effect size we found in a previous synthesis project investigating reduced depressive symptoms in 19 prevention programs with a two year follow-up (see Figure 5 in the following reference).[46] We expect this effect size given that we are now including youth who are elevated on depressive or anxiety symptoms or have a history of suicide ideation or attempts. For suicide ideation, our growth models for repeated binary measures indicates we will have over 70% power to detect a yearly odds ratio (OR) reduction of 0.50 when the prevalence of ideation at baseline is 0.12 (or 12%). For suicide attempts, the baseline prevalence we have observed previously is 0.086 (or 8.6%), and this provides 55% power to detect an OR of 0.5. These ORs and prevalence rates were taken from our most recent Familias Unidas trials using the subset of youth with poor family communication, matching our inclusion criteria. For suicide attempts, if we obtain an OR of 0.4, we would have 71% power to detect this change from baseline for attempts and 88% power for ideation. We will analyze impact on K-CAT MDD as well as use missing data and a two-stage design to calibrate these findings against K-SADS MDD, see section 4.4.). Thus, we have sufficient power to detect a large effect on suicide outcomes over time. For the power of detecting a mediation effect, we take as an example 6-month family communication mediating 18-month depressive symptoms. If both the a and b estimators have 80% power of rejecting their nulls, then the product has a 64% power of rejecting the mediation pathway as being significant. If each a and b estimators have 85% power, the product has 72% power of rejection. Additional details are provided in Clinical Trials Section 4B.

Post-hoc Analyses. A number of post-hoc analyses will be conducted. First, because we will also measure past 90 day frequency of alcohol and cigarette use, we will examine condition effects on cigarette and alcohol use. This is important because should the intervention have an impact on these outcomes, this would increase the likelihood that the U.S. Preventive Services Task Force, which makes recommendations about preventive services to integrate within primary care, would recommend eHealth Familias Unidas Mental Health as an intervention which should be integrated into primary care settings. Such a policy change would greatly enhance the sustainment of eHealth Familias Unidas Mental Health because an A or B rating would provide reimbursement for Medicaid and private insurance. We also recognize that there may be subgroups of Hispanic youth, such as LGBTQ, US born and acculturated Hispanic youth, girls, youth who experience ethnic bullying, families with high acculturative stress and low SES families who may be at higher risk for internalizing symptoms and suicide. As such, we will analyze youth differences in outcomes by these high-risk subgroups as well as differences in the mediation pathways towards reduced mental health problems and suicide related behavior and ideation.

Risks to Subjects*

We do not expect that there will be any negative consequences from participating in this study. However, the sensitive nature of some intervention topics (e.g. sexual behaviors and drug use) may be embarrassing and/or distressing to some participants. It is also possible that they may feel tired after answering the questionnaires, or made uncomfortable by the questions.

If a parent or adolescent reports elevated levels of depression, or if an adolescent reports incidence of suicidality, he or she will be directed to speak with a facilitator for further assessment. If necessary, a referral will be made for additional services.

Video session recordings will be saved onto secure university servers. All data with identifiable information such as names, date of birth, etc., will be numerically labeled and kept separate from assessment data. Only PIs and authorized study personnel will have access to data files and participant linking lists. The risks associated with gathering information from participants by properly trained and supervised professional research staff are low and include risks of loss of privacy and transient psychological distress. The research team has extensive experience in conducting intervention and assessment protocols that involve underserved populations as well as highly confidential personal information. Quality assurance protocols, emergency procedures, and crisis intervention, and referral procedures have been established in previous trials and can be modified as needed for the proposed study.

We will conduct data analysis of deidentified clinical notes that are taken as part of our existing procedures to triage adolescents who have reported incidence of suicidality on the assessment battery. This is data that has resulted from existing study procedures; there are no new study procedures being added. When adolescents have reported incidence of suicidality and they have been triaged by a study team member, documentation of these incidents will be the source of data. We will analyze the data to extract common themes that may emerge in a summary format without identifiable participant characteristics and/or information.

8) Potential Benefits to Subjects*

There may be direct benefits to participating in this study. Adolescents may be less likely to use drugs and engage in risky behaviors such as having sex without a condom. Also, adolescents may have decreases in depression and anxiety symptoms and suicide thoughts or acts. Additionally, families may communicate more effectively. This study also has the potential to provide knowledge regarding how to best implement evidence-based interventions in primary care settings where it can be offered to Hispanic youth, a population with historically low access to preventive interventions.

9) Vulnerable Populations*

The research involves children under the age of 18. To avoid coercion or undue influence, the adolescents will assent and complete assessments separate from their primary care giver. In case the adolescent does not want to assent, the family will be told they did not meet one or more of the inclusion/exclusion criteria but the facilitator will not reveal to the parent that the child did not assent (to avoid any consequences to the child).

10) Setting

Participants will be recruited from primary care clinics in Florida. The sites are heterogeneous and consist of federally qualified health centers, academic primary care clinics located within the University of Miami's Health District, South Florida's only public health hospital, and private community clinics. The clinics include: Agape Network; Banyan Health System; Baptist Health System; Borinquen Medical Centers (Biscayne and 8th street); Bravo Pediatric Clinic; Broward Health; Care Resources; Community Health of S. Florida; Community Health of South Florida-Homestead;

UHealth Pediatrics at Kendall; Jessie Trice Community Health System; Nicklaus Children's Hospital; Pediatric Mobile Clinic; Prime Care Health; South Florida Pediatric Partners; UHealth Pediatrics at PAC; UM Mailman Center for Child Development.

The personnel at the clinics will deliver the intervention, however, these personnel are research participants and are delivering the intervention as part of their research activities on the research study. Because these clinic research participants are conducting research activities on behalf of the study and due to their study involvement, the actual clinic itself is not engaged in the research.

11) **Resources Available**

Dr. Guillermo Prado, Contact *Principal Investigator* is Professor of Nursing and Health Studies, Public Health Sciences, and Psychology at the University of Miami. He is considered one of the top experts in the development and evaluation of culturally specific family-based preventive interventions for Hispanic youth. His research has led to more than 150 peer reviewed publications, including the major publications of Familias Unidas randomized clinical trials (e.g., Prado et al., 2007; 2011; 2012; Estrada et al., 2017). Additionally, in the past twenty years, he has been (or currently is) Principal Investigator, Co-Investigator, or mentor of more than $75 million dollars of NIH and CDC funding. Further, Prado is currently president for the Society of Prevention Research, the flagship professional organization for prevention science. As one of the PIs of the proposed study, Prado will be responsible for providing scientific leadership as well as the day-to-day management of the project including all personnel and research activities. Prado will also work with Co-PI Brown, who will have the lead responsibility in the analyses. Prado will be responsible for overseeing the recruitment and assessment process (along with Estrada), and triaging issues of consent and confidentially, including relations with the IRB. Prado will establish and maintain relationships with the participating clinics. Finally, Prado will co-lead, along with Brown, the execution of all scientific, managerial, and fiscal aspects of the project. As contact PI, Prado will handle relationships with the National Institute of Mental Health.

C. Hendricks Brown, MPI, PhD, is Professor in the Departments of Psychiatry and Behavioral Sciences, Preventive Medicine, and Medical Social Sciences. He is Director of the NIDA-funded Center for Prevention Implementation Methodology for Drug Abuse and HIV and the Prevention Science and Methodology Group. In his role as MPI he will lead the team in finalizing the research design and specifying the statistical analyses for evaluation. He will also participate in all the major decisions of the trial, including adapting new components into eHealth Familias Unidas, tracking youth, families, clinical staff, and site coordinators participation and activities, participating in DSMB meetings, and publications and presentations. To coordinate work, Brown will travel to Miami 3 times a year.

Dr. Tatiana Perrino, *Co-Investigator* , is an Associate Professor in the Department of Public Health Sciences at the University of Miami Miller School of Medicine. Perrino, a licensed clinical psychologist, is a prevention scientist whose research has focused on promoting mental and behavioral health among disadvantaged, Hispanic youth. Perrino was an Investigator on a recently completed NIMH-funded data (PI, C Hendricks Brown) on the prevention of adolescent depression, a study that examined effects, moderators and mediators across 19 youth depression trials (Howe, Pantin & Perrino, 2018). Perrino has

led the articles on the crossover effects of Familias Unidas on internalizing symptoms (Perrino et al., 2015; 2016a; 2016b), as well as important studies on the promotion of mental health equity (Brown et al., 2018; Perrino et al., 2014; 2015; 2019). Perrino was invited to speak at a National Academies of Sciences, Engineering and Medicine webinar on preventive interventions for youth depression, and to review the Academies' national workshop proceedings on behavioral health equity for children, families and communities. Perrino has collaborated extensively with Prado and Brown over the past 15 years. For this study, Perrino will collaborate with Prado, Brown, and Estrada on the adaptation of eHealth Familias Unidas-Mental Health. Perrino's expert knowledge will inform intervention modifications including session content, updating of the intervention manual, and updating of the supervision guidelines for the newly adapted intervention. Perrino will also participate in the interpretation of study findings and the writing of study publications.

Other personnel include Dr. Yannine Estrada, Project Director for the study and an Assistant Scientist at the University of Miami School of Nursing and Health Studies. Estrada, a licensed counseling psychologist, has extensive experience in directing randomized controlled trials. In fact, she has been responsible for directing the four most recently completed studies on Familias Unidas studies and has been responsible for overseeing all aspects of data collection. Estrada also led, in conjunction with Prado, the development of eHealth Familias Unidas and is, therefore, well versed in the technological logistics, background informatics, and process of the intervention, which will be crucial for executing modifications.

Estrada has a long track record of working with Prado which extends over 10 years. For this study, Estrada will collaborate with the senior investigative team in the adaptation of eHealth Familias Unidas along with planning the outcome analyses and preparing manuscripts for publication. Estrada will also be responsible for coordinating the research and research-clinical interface aspects of the study.

Ms. Maria Tapia, Clinical Trainer and Supervisor is a Licensed Clinical Social Worker with over 20 years of experience working with Hispanic families. She is trained in delivering family-based interventions, including eHealth Familias Unidas, to Hispanic parents and their youth. Tapia has engaged over 80% of Hispanic parents into the Familias Unidas intervention over the past 15 years. She also has extensive experience training and supervising the Familias Unidas model. For the proposed study, Tapia will be responsible for training and supervising the primary care facilitators in the delivery of the intervention. She will also conduct the supervision meetings with the primary care facilitators and will help them problem solve potential barriers of intervention engagement and retention. Additionally, Tapia will handle any adverse or serious adverse events that may occur during the intervention sessions. Tapia will also provide input regarding development of the new intervention modules in year 1.

Assessors will be responsible for screening potential study participants and conducting the parent and youth assessments over the four years. Assessors will conduct over 7,000 assessments over the four years (i.e., 468 youth and 468 parent assessments per assessment timepoint x 4 assessment timepoints). They will also be responsible for scheduling these assessments. Finally, assessors will also be responsible for maintaining cordial contact in between assessment timepoints, in order to maximize retention rates at assessments.

Other study personnel, such as student, research assistants and facilitators will be trained in study

operating procedures, including consent/assent. All study personnel members are CITI trained and are adequately informed about the protocol, research procedures, and their duties and functions. Trainings will be held for study personnel members before the beginning of the study in: protocol, research procedures, and assessments. Study personnel will be asked to role play procedures at the training. If more training is needed, they will be trained again throughout the study. All of the study key personnel have been involved in NIH funded studies and are all familiar with the recruitment/study procedures as those being proposed. Regardless of experience, trainings will still be held by the lead clinical supervisor, project director, and principal investigators to assure all study personnel members are adherent to the study procedures. Weekly meetings will also be held to discuss study updates, issues or concerns.

Scientific Advisory Board (SAB): The SAB is comprised of senior advisors who will advise Prado, Brown, and Perrino on the adaptation of eHealth Familias Unidas for mental health and on the implementation of the intervention in primary care settings. The SAB members include:

Dr. Viviana Horigian, a board certified psychiatrist, with over 15 years of experience in working with Hispanic families is an Associate Professor of Public Health Sciences. Dr. Horigian has been the Executive Director of the Florida Node Alliance of the National Drug Abuse Treatment Clinical Trials Network for the past 15 years. In this role, she has led or co-led studies in primary care settings and has established relationships with some of the clinic sites being proposed. She has also co-led implementation studies as part of the Clinical Trials Network. Along with the other two SAB members, Horigian will consult on the adaptation of eHealth Familias Unidas as well as on its integration and sustainment in primary care.

William Beardslee, academic chairman of the Department of Psychiatry at Children’s Hospital in Boston and the Gardner Monks Professor of Child Psychiatry at Harvard Medical School

Jami Young, Director of Psychological Research, in the Department of Child and Adolescent Psychiatry and Behavioral Sciences at Children’s Hospital of Philadelphia.

12) **Prior Approvals**

None.

13) **Recruitment Methods**

Screening will take place in pediatric care clinics. Screening will be included into the patient check-in process. Parents and adolescents will complete the screening separately on tablets provided by the research team. REDCap programming will be used to determine whether the participant meets the screening criteria. Families who meet study inclusion/exclusion criteria will automatically be connected via protected videoconference software to the on-call study team via iPad to completethe e-consent process. If a videoconference call is not possible, arrangements will be made at a convenient time for the participant. The team member will explain the study to eligible participants and complete the e-consent process. Families who enroll will then be sent a link to complete the assessment battery via iPad while they wait for the physician. The clinic staff member will thank the study participant for enrolling in the study and will make a hand off to the physician. We will screen and enroll youth with poor family communication or elevated depressive or anxious symptoms, as well as those with a history of

suicide ideation or attempts (see inclusion criteria).

Participants will be assessed at baseline and 3-, 6-, and 18- months post-baseline. We have budgeted the following amounts for participant parent incentives: T1=$40, T2=$45, T3 = $50 and T4= $55. Adolescents will receive two movie tickets and $20 for each completed assessment. The participant incentive cost will reimburse study participants for time and effort to participate in the baseline and each of the follow-up assessment activities.

As an additional form of recruitment, clinic doctors will also provide families with a note where they recommend the program. If the family is interested in participating, they will provide their contact information and a research team member will call them to explain the study. We will also distribute flyers in the clinics to recruit participants. These flyers will contain a QR code that can be scanned by potentially interested participants. Once scanned, participants will be able to provide their contact information so that they can be reached and given more information about the study.

Additionally, we will do the following:

Research study staff will request permission from each clinic, at UM clinics only and not at any external clinics, to have access to their scheduling system. On a daily basis, research study personnel will access data on which individuals have a scheduled appointment. No health information will be available to research study staff, only parent name, child age and whether there is an appointment scheduled. Study staff will use the study's screening form to screen these individuals, at the clinic, to see which meet criteria. Study staff will then go to waiting room to recruit those that meet criteria.

Registry Recruitment –We will also collaborate with the University of Miami's Clinical & Translational Science Institute (CTSI) Consent to Contact registry to identify potential participants. They will provide us with contact information for patients who have provided permission to be contacted by study projects. We will send out a general recruitment flyer, email, or text (depending on a participant's preferred choice of contact) to these families and we will invite them to complete the REDCap screening survey and consent if they are interested in the eHealth Familias Unidas for Mental Health project. A partial waiver of HIPAA is requeted to utilize the Consent to Contact database

14) **Confidentiality**

The issues surrounding confidentiality are of supreme importance and sensitivity because personal information will be obtained from the adolescent and their family members. Participants sign a statement attesting to their understanding that the information they provide will be held as personal and confidential. Consent forms clearly state the right to refuse participation at any time. In addition, because this project involves the collection of mental health, drug use and sexual behavior information, to strengthen the security of our records in relation to local and state courts, a Confidentiality Certificate under section 502C of Part E, Title II of the Comprehensive Drug Abuse Prevention and Control Act of 1970, Public Law 91-513, has been obtained. This Certificate has been utilized in previous prevention studies and has been instrumental in maintaining the privacy/confidentiality of sensitive client information.

With this Certificate, the researchers cannot be forced, even by a court order, to share research information that may identify the participants in any civil, criminal, administrative, legislative, or other proceedings in any court. The researchers will use the Certificate to resist any demands for information that would identify the participants, except to prevent serious harm to them or others, and as explained below.

The participants should understand that a Certificate of Confidentiality does not prevent them, or a member of their family, from voluntarily releasing information about themselves, or their involvement in this study. If an insurer or employer learns about their participation, and obtains participant's consent to receive research information, then we may not use the Certificate of Confidentiality to withhold this information. This means that the participants and their family must also actively protect their own privacy. Disclosure will be necessary, however, upon request for the purpose of audit or evaluation, and is limited only to Department of Health and Human Services employees involved in the review. The participant should understand that we will in all cases, take the necessary action, which may include reporting to authorities, to prevent serious harm to themselves or others.

(A) Names;
(B) All geographic subdivisions smaller than a State, including street address, city, county, precinct, zip code, and their equivalent geocodes, except for the initial three digits of a zip code if, according to the current publicly available data from the Bureau of the Census:
(1) The geographic unit formed by combining all zip codes with the same three initial digits contains more than 20,000 people; and
(2) The initial three digits of a zip code for all such geographic units containing 20,000 or fewer people is changed to 000.
(C) All elements of dates (except year) for dates directly related to an individual, including birth date, admission date, discharge date, date of death; and all ages over 89 and all elements of dates (including year) indicative of such age, except that such ages and elements may be aggregated into a single category of age 90 or older;
(D) Telephone numbers;
(E) Fax numbers;
(F) Electronic mail addresses;
(G) Social security numbers;
(H) Medical record numbers;
(I) Health plan beneficiary numbers;

(J) Account numbers;<br>*(K) Certificate/license numbers;*<br>*(L) Vehicle identifiers and serial numbers, including license plate numbers;*<br>*(M) Device identifiers and serial numbers;*<br>*(N) Web Universal Resource Locators (URLs);*<br>*(O) Internet Protocol (IP) address numbers;*<br>*(P) Biometric identifiers, including finger and voice prints;*<br>*(Q) Full face photographic images and any comparable images; and*<br>*(R) Any other unique identifying number, characteristic, or code*

Protected Health Information (PHI) is defined under HIPAA in 45 CFR § 160.103. The following list of identifiers of an individual, or of relatives, employers, or household members of the individual, are defined under HIPAA in 45 CFR § 164.541:

Choose the statements below that are applicable to this research:

15(a). ☐ Data will be collected from the EMR or subjects at UHealth or JHS. *If checked, answer the following:*

☐ Research Subjects will sign a HIPAA Authorization before the research will collect this data.

☐ Research Subjects will not sign a HIPAA Authorization for this data collectionand the research is requesting a waiver of HIPAA authorization from the IRB. (Complete Section 17 below)

15(b). Data collected:

☐ Will not include Protected Health information or Personally Identifiable Information

☐ Will include Protected Health information or Personally Identifiable Information

15(c). How will the research store the data?

☐ On a University of Miami electronic device (e.g. encrypted, password-protected computer)

☒ On a cloud-based storage system that is approved by the University of Miami

☐ Other, specify: Click here to enter text.

Select one of the following:

☐ The Principal Investigators (and/or Study Team members) will record (e.g. write

down, abstract) data acquired in a manner that **does not include any** indirect or direct

identifiers (listed in the instructions for Section 15 of this protocol), and the recorded data will not be linked to the individual's' identity.

OR

☒ The Principal investigators (and/or Study Team members) will record (e.g. write down, abstract) the data collected in a manner that does not include any direct identifiers (see list in the instructions for Section 15 of this protocol) of any subject. Instead, the Principal Investigator and/or Study Team members will assign a code (that is not derived in whole or in part from any direct or indirect identifiers of the individual) to each study subject and link the code to the study subject's identity. **The link to each subject's identity and/ or other identifiable information will be maintained on a document separate from the research data.**

Biospecimens

☒ Not applicable. No biospecimens will be collected

☐ *Bio*-Specimens obtained for this research will be stored without any direct or indirect identifiers.

☐ *Bio*-Specimens obtained for this research will be stored in a de-identified coded manner.

☐ When required to transport data or bio-specimens for this research, the research team will transport the data and bio-specimens in a de-identified (or anonymous) manner with a link to the individual subject's identity maintain separately from the data and/or bio-specimen.

15d. Jackson Health System additional requirement

☒ This section is not applicable because the research is not collecting health information from JHS under a waiver of authorization (without obtaining a HIPAA authorization from the participant)

If health information, including Protected Health Information and/or Personally Identifiable Information are collected from JHS without a signed authorization from the subject (with a waiver of authorization from an IRB or Privacy Board), you must agree to the following:

☐ JHS data, including Protected Health Information (PHI) and/or Personally Identifiable Information (PII), acquired from JHS for this research with a waiver of the requirement for an authorization under HIPAA shall only be stored on the secured JHS SharePoint environment made available by JHS. I and the Study Team members shall not copy or store the JHS sourced personally identifiable information (PII), including protected health information (PHI) data to any other system, including any systems maintained or provided by the University of Miami. I and the Study Team shall only copy or transfer JHS-sourced data that has been

properly de-identified in accordance with all requirements contained in the HIPAA Rules by removing all of the identifiers listed in the instructions for Section 15 of this protocol.

If the data obtained for this research will be acquired from a retrospective “chart review” involving health information from JHS with a waiver of authorization (without obtaining an signed HIPAA authorization from the subject) then the data and the link and/or key to each subject’s identity shall only be maintained in the secure JHS SharePoint environment made available by JHS.

15) Provisions to Protect the Privacy Interests of Subjects

During the recruitment and electronic consent/assent process, and prior to the assessments, participants are told that information is private, confidential and only accessed by specific study personnel. Participants are given a case ID to further protect their identity. Participants are free to ask any questions, and the consent form has the contact information of the Principal Investigator should they have further questions and the phone number to the University of Miami’s Human Subjects office.

In the informed consent forms, participants will be advised that we may use de-identified survey data collected for research purposes and provide the de-identified survey data to other researchers for further analyses. Participants’ information will be removed from the survey data. Once the identifiers have been removed, we will not ask for participants' consent to use or share their survey data for research.

We do not expect for this study to create more than minimal risk for the participating families. Yet, the study will be monitored by the study investigators and/or sponsors. In addition, a formally constituted Scientific Advisory Board will help monitor the study if additional advice is needed.

16) Waiver of Authorization for Use and Disclosure of Protected Health Information (HIPAA)

If the research team will access patient medical records or other identifiable health information for this research without or prior to obtaining a signed HIPAA authorization from the subject or the subject’s legally authorized representative (LAR), you must obtain a waiver of the requirement for written authorization from the patients to access their medical records.

Confirm that you will destroy the Protected Health Information (PHI) you and/or your Study Team acquire receive from JHS and/or UHealth at the earliest opportunity.

X I confirm

Confirm that the Protected Health Inform (PHI) you acquire from JHS and/or UHealth will not be re-used or disclosed to any other person or entity, except as required by law or for authorized oversight of the research study or for other research for which the use or disclosure

of PHI is permissible.

X I confirm

If you are collecting health information from JHS under a waiver of authorization, you must read the paragraph below and sign the signature block to indicate your agreement:

Notwithstanding the preceding "I confirm" statements above, I agree that neither I nor any member of the study team listed on the IRB submission for this Protocol shall ever re-use or re-disclose any of the information acquired from Jackson Health System in any format, whether **identifiable or de-identified,** to any individual or entity without first obtaining written permission from Jackson Health System, even if such re-use or re-disclosure is permissible by law (e.g., HIPAA).

____

PI Signature Date

17) **Consent Process**

The consent process will take place on tablets. Screening will be included into the patient check-in process. Families who meet study inclusion/exclusion criteria will automatically be connected via protected videoconference software to the on-call study team via iPad to complete the e-consent process. A study team member will present and read the electronic consent forms to the caregivers who are eligible to participate based on the inclusion and exclusion criteria. The consent form will be an electronic document bearing the HSRO approval stamp/watermark. The consent and assents will be available in English or Spanish. We will use the same procedures for translating and back translating as in previous studies. After the caregivers understand the study and after all questions have been answered to their satisfaction, individuals who elect to participate in the study will be asked to sign the consent form. The consent process will last approximately 30 minutes, but it may take caregivers a little less or a little more depending on how clearly they comprehend the study procedures.

If a parent is not available to complete consent, a legal guardian is allowed to provide permission for the adolescent to assent. A legal guardian is a step mother or step father, or another person who has obtained legal rights to the child. During the recruitment screening process, study personnel will determine if the primary caregiver is a legal guardian if they demonstrate the correct paperwork.

Since recruitment will take place at different clinics, it is a possibility that potentially eligible families will visit the clinic when the research team is not available to complete the online consent process with the family. If the clinic personnel identifies such a family, they will be able to provide them with a flyer about the study and information on how to contact the research team. If the family is interested and contacts the research team, they will be contacted by the research team so that the

family can be informed about the study and conduct the consent, assent and baseline assessment, if the family is interested in being in the study. Adolescents will only be contacted to participate after the primary caregiver has given consent.

If other family members (adults or minors) want to participate in the virtual family sessions. They will be required to sign an electronic secondary informed consent through REDCap. (1) For other adults, **the secondary adult consent**. (2) For minor(s) to have signed, from the parent or legal guardian, a **secondary adult consent for a minor**, and the **secondary minor assent for the other minor**(s).

18) **Process to Document Consent in Writing**

Consents will be stored electronically in secure University of Miami servers. Information for de-identified participants who do not wish to participate in the study and provided verbal consent/assent to answer the questions for non-participating families will be kept in secure University of Miami servers.
