# Supplementary material for: eHealth Familias Unidas Mental Health: Protocol for an effectiveness-implementation hybrid Type 1 trial to scale a mental health preventive intervention for Hispanic youth in primary care settings
Source: PLoS One. 2023 Apr 18;18(4):e0283987. doi: 10.1371/journal.pone.0283987 (PMC10112791; doi:10.1371/journal.pone.0283987)
Supplement: S3 File — (PDF) [file pone.0283987.s004.pdf]

Permission to Take Part in a Human Research Study—SBS

Parent English

Title of Study: Scaling a Parenting EBI for Latinx Youth Mental Health in Primary Care
Principal Investigator: Guillermo "Willy" Prado, PhD
Department: School of Nursing and Health Studies
Phone Number: 305-284-2002
Email Address: gprado@miami.edu
Study Contact Name: Yannine Estrada, PhD
Study Contact Telephone Number: 305-284-6191
Study Contact e-mail: yestrada@miami.edu

Sponsor: National Institute of Mental Health

You and your child are being asked to take part in a study by the University of Miami School of Nursing and Health Studies. You are being asked because you are Hispanic and have a child between the age of 12-16. We will ask about 468 families like yours. The purpose of this study is to see if an online delivered program, *eHealth Familias Unidas Mental Health,* improves communication and adolescent mental health and decreases drug use and sexual risk behaviors among Hispanic youth. Also, in this study we will explore if the program is sustainable by primary care clinics. The program will be delivered by personnel from your child's primary care clinic.

What happens if I say yes, I want to be in this research?

If you agree to be in this research, you and your adolescent will be asked to complete surveys. You and your adolescent may also be asked to participate in the online program, eHealth Familias Unidas for Mental Health. Whether you are asked to participate in the program or not will depend on when your child's primary care clinic offers the program. Below we describe the program and surveys.

Program: If the program is being offered at the time that you enroll in the study, the program will be delivered through our website, and you may be asked to access the website in the clinic today. If the program is not being offered at the time you enroll in the study, you will only complete the surveys.

- You may be asked to watch nine videos on our website. Each video is approximately 30 minutes.
- Your adolescent will be asked to watch one video from our website that lasts about 30 minutes.
- Someone from your clinic will arrange four video calls, or "family sessions," with you and your adolescent.
    - Each family session will be about 45 minutes and you will have a chance to talk to your child about what you learned from watching the videos on the website and how to better communicate.
    - **Video Recordings**: These sessions will be video recorded to see how the facilitator interacts with you. If you or your adolescent do not want to be video recorded, you can sit outside of the site of the webcam. If you do not wish to be video recorded, your family will not be penalized in any way. By consenting to be part of this study, you agree that the photographs, film, video or audio recordings can be used for the purpose of research, teaching, training, and presenting at scientific conferences. All video recordings will be electronically stored in secure University servers. Your video recordings will only be accessed by study staff. The recordings are not labeled with your name but with identifiers assigned to you at the beginning of the study.

- The videos and video calls will be completed at home, or another private location, over the next thirteen weeks.

Surveys:

- If you agree to join this study, you will be asked about your family, child, and about yourself. The topics asked include basic facts about where you come from, your age, income, education, and contact information. We will ask about your and your child's mental health. We will ask you and your child whether you smoke, drink alcohol, or use illicit drugs. Your child will be asked about the relationship he or she has with his/her family, and yourself. Finally, we will also ask your child questions about his or her sexual behavior and sexual orientation.
- You/your child do not have to answer any questions that you do not want to answer. If you or your child decide not to answer questions, nothing bad will happen to you.
- You and your child will complete the survey 4 times: when you enroll, and 3, 6, and 18 months after you enroll. You may complete the surveys at the clinic or via a link that we email to you. We may contact your child by email to complete the follow-up surveys.
- Your and your child's survey will take about 30-45 minutes.

What should I think about before I enroll in this research?
You should ask any questions you may have and obtain answers before you decide.

Do I have to be in this research?

No. Your and your child's participation in this study is voluntary. You and your child do not have to be in this study if you do not want to, and you can leave the study at any time. You and your child will not lose any services, benefits, or rights that you would normally have if you chose not to be in the study or if you leave the study early. The investigator reserves the right to remove you and your child without your or your child's consent at such time that they feel it is in the best interest for you and your child.

Is there any way being in this study could be bad for me?

We do not expect that there will be any harm to you or your child for being part of this study, but you may be bothered by some of the topics of the program or survey, and/or you might also feel tired after answering the surveys.

What are the benefits to being in this study?
You may not benefit from being part of this study. However, you, your child, and/or your family may be able to communicate better, may be less likely to smoke, drink alcohol, use drugs, or engage in unsafe sexual behavioral. You and your child may also be less likely to have symptoms of depression, anxiety, and/or suicide thoughts or behaviors.

What happens to the information collected for the research?

What you tell us will be kept private to the extent allowed by law. We will not reveal any of your survey answers to the medical team in the primary care setting, and nothing you tell us will appear in your child's medical records. Also, to protect your child's privacy, we will not tell you what he or she says in his or her responses to our surveys. The researchers will keep all study records, including any codes to your data, in a secure location. Data that is not electronically collected will be kept in a locked cabinet. Research records will be labeled with a code. A master file that links names and codes will be

maintained in a password protected university server. All electronic files containing identifiable information will be password protected. Only the members of the research staff will have access to the passwords. We may use de-identified survey data collected from you (and/or your adolescent) for research purposes and provide the de-identified survey data to other researchers for further analyses. All information that can identify you will be removed from the survey data. Once identifiers have been removed, we will not ask for your consent to use or share your (your adolescent's) survey data for research. At the end of this study, the researchers may publish their findings. Information will be presented in summary format, and you will not be identified in any publications or presentations.

Despite taking all of the safety measures listed above, there is always the risk that your privacy will be broken.

Your information may be looked at and/or copied for research or regulatory purposes by:

- The sponsor, if any;
- Department of Health and Human Services (DHHS);
- other government agencies;
- other University of Miami employees for audit and/or monitoring purposes; and
- other organizations collaborating in the research

A Certificate of Confidentiality (CoC), issued by the NIH, covers this research. A CoC helps protect your identifiable information.

A CoC protects your private information from all legal proceedings. Unless you consent, information from this research study that identifies you will not be shared outside this research except as described above.

- No one can be forced to share your identifiable information for a lawsuit.
- Your information can't be used as evidence even if there is a court subpoena.

The CoC does not prevent some disclosures.

- The researchers can't refuse requests for information from those funding this research. The National Institute of Mental Health may need information to assess this project.
- You can still share information about yourself. You can also freely discuss your involvement in this research, but this is your choice. The information you share will no longer be protected by the CoC.
- The researchers must disclose things required by law. This includes suspected child abuse and neglect, harm to self or others.

This trial will be registered and may report results on www.ClinicalTrials.gov, a publicly available registry of clinical trials.

Sometimes people in our studies move and we can't find them. Therefore, we will ask you to give us the names and telephone numbers of three people who will always know how to reach you, in case we lose contact with you. We will never share anything you tell us with these people.

Payment

If you agree to be in this research study, we will pay you $40 for the first survey, $45 for the second, $50 for the third, and $55 for the last for your time and effort. Your child will receive two movie tickets and $20 for each survey for his/her time and effort.

Your information (both identifiable and de-identified, as relevant) may be used to create products or to deliver services, including some that may be sold and/or make money for others. If this happens, there are no plans to tell you, or to pay you, or to give any compensation to you or your family. Any data obtained for the purposes of this study become the exclusive property of the University of Miami. The University of Miami may retain, preserve, or dispose of this data for research which may result in commercial applications.

Who can I talk to?

If you have questions, concerns, or complaints, or think the research has hurt you, talk to the research team at 305-284-2002 with Dr. Guillermo "Willy" Prado, the person leading this research

This research has been reviewed and approved by an Institutional Review Board ("IRB"). The Human Subject Research Office (HSRO) provides administrative support to the University of Miami's IRBs. Please call the HSRO at 305-243-3195 if you are a participant in any research being conducted by UM, and:

- Your questions, concerns, or complaints are not being answered by the research team.
- You cannot reach the research team.
- You want to talk to someone besides the research team.
- You have questions about your rights as a research subject.
- You want to get information or provide input about this research.

PARTICIPANT'S STATEMENT/SIGNATURE

- *I have read this form and the research study has been explained to me.*
- *I have been given the chance to ask questions, and my questions have been answered. If I have more questions, I have been told who to call.*
- *I agree to be in the research study described above.*
- *I will receive a copy of this consent form after I sign it.*

____

Printed Name of Child

____

Name of Parent or Primary Caregiver (First, Last)

______________________________________ ________________

Signature of Parent or Primary Caregiver (First, Last) Date

____
Printed Name of Person Obtaining Consent

____ ________________
Signature of Person Obtaining Consent Date

Future Studies

We may conduct additional studies related to the study described in this form. If we do conduct these studies, we will contact you in the future to see if you would like to be part of the future study. Your participation in this study does not depend in any way on your participation on any future study. If we have difficulty finding you, we may call your contact persons to find out where you are.

I agree to be contacted after the study ends to be asked to take part in future studies.

□ Yes □ No

I agree to allow my child (if he/she is under the age of 18) to be contacted after the study ends to be asked to take part in future studies.

□ Yes □ No

____
Youth Name (First, Last)

____
Name of Parent or Primary Caregiver (First, Last)

_______________________________________ ________________
Signature of Parent or Primary Caregiver Date

____
Name of Person Obtaining Consent/Assent (First, Last)

_______________________________________ ________________
Signature of Person Obtaining Consent/Assent Date
